# Measuring Health System Resilience During the COVID‐19 Pandemic Using Dynamic Indicators of Resilience Based on Sick‐Leave Data

**DOI:** 10.1002/hsr2.70789

**Published:** 2025-06-23

**Authors:** Tom H. Oreel, Sophie Hadjisotiriou, Vítor V. Vasconcelos, Vincent A. W. J. Marchau, Etiënne A.J.A. Rouwette, Rick Quax, Vittorio Nespeca, Jannie Coenen, Hubert P. L. M. Korzilius, Heiman Wertheim, Marcel G. M. Olde Rikkert

**Affiliations:** ^1^ Department of Geriatrics, Department of Geriatric Medicine Radboud University Medical Center Nijmegen The Netherlands; ^2^ Computational Science Lab, Informatics Institute University of Amsterdam Amsterdam The Netherlands; ^3^ Institute for Advanced Study University of Amsterdam Amsterdam The Netherlands; ^4^ Center for Urban Mental Health University of Amsterdam Amsterdam The Netherlands; ^5^ Institute of Management Research Radboud University Nijmegen The Netherlands; ^6^ Department of Business Administration, Methodology Department Radboud University Nijmegen The Netherlands; ^7^ Department of Medical Microbiology Radboudumc Center for Infectious Diseases Radboudumc The Netherlands

**Keywords:** COVID‐19, epidemiology, healthcare systems, resilience

## Abstract

**Background and Aims:**

Healthcare system resilience is generally understood as the capacity of a healthcare system to prepare, withstand, and adapt to disruptive health events while maintaining the continuity and quality of essential health services. So‐called dynamic indicators of resilience (DIORs) allow us to examine resilience by analysing patterns of functioning of the healthcare system in time series data. The aim of this study was to examine whether DIORs can be estimated from time series data of the functioning of the Dutch healthcare system before, during and after the COVID‐19 pandemic, and whether these DIORs are indicative of the resilience of the Dutch healthcare system during the COVID‐19 pandemic.

**Methods:**

To select a measure of healthcare functioning, healthcare experts completed a questionnaire in which they selected the five most relevant indicators of healthcare availability (table s14). Based on the questionnaire results and datasets available, time series data of sick‐leave absenteeism rates among Dutch healthcare workers before, during and after the COVID‐19 pandemic were used to quantify the functioning of the Dutch healthcare system. DIORs were estimated using moving window techniques on the time series data of each healthcare sector, each safety region in the Netherlands, and all healthcare sectors and safety regions in the Netherlands combined.

**Results:**

Short‐term sick‐leave increased from 3.2% to 4.5% and long‐term from 3.0% to 4.0% post‐pandemic (*p* < 0.001). DIORs showed significantly increasing autocorrelation during the pandemic (Kendall's *τ* = 0.46–0.52), indicated an increased loss of resilience of the Dutch healthcare system as the COVID‐19 pandemic progressed. Trends were consistent across healthcare sectors but varied across regions, with some regions showing stable or improving resilience.

**Conclusion:**

Our results indicate that DIORs, estimated from time series data of sick‐leave absenteeism rates among healthcare workers in the Netherlands during the COVID‐19 pandemic, potentially provide useful insights into healthcare system's resilience during and following disruptive health events, such as the COVID‐19 pandemic.

## 1 Background and Aims

### 1.1 Healthcare System Resilience During the COVID‐19 Pandemic

The COVID‐19 pandemic heavily disrupted healthcare systems worldwide, highlighting the importance of healthcare systems resilience. In general, resilience refers to a system's ability to maintain normal functioning in the face of change [1]. While the concept of resilience is used in different fields, its application to healthcare systems is relatively new [2, 3]. *Healthcare system resilience* is generally understood as the capacity to prepare, withstand, and adapt to disruptive health events while maintaining the continuity and quality of essential health services [3, 4, 5, 6]. By continuously monitoring healthcare system resilience, policymakers gain valuable insights into the healthcare system's ability to withstand and respond to disruptive health events. Such information helps policymakers to make informed decisions to maintain continuity and quality of essential health services in the face of disruptive health events, being new pandemics or other disasters [7].

### 1.2 Measuring Healthcare System Resilience

Previous research on healthcare system resilience has so far mostly remained theoretical and previous attempts to quantify healthcare system resilience do not take into account the interconnected nature of the different subsystems in healthcare (e.g., between public health services, primary care, and intensive care [8]), nor healthcare with other societal systems (e.g., healthcare and education or healthcare and business [9, 10]). Considering healthcare systems as complex systems of various interactions and relationships among important factors (e.g., absence health professionals, available ICU beds) that together determine its overall functioning allows us to apply principles of complexity science to quantify their resilience [11]. One principle of complexity science, “critical slowing down” seems particularly promising in the context of healthcare system resilience, as it has proved to be a measure of resilience in other complex systems and it can be inferred from time series by examining the recovery rates from natural occurring stressors [12, 13, 14]. For example, as a system becomes less resilience it requires more time to recover from stressors, resulting in slower fluctuations of the functioning of the system, which can be seen in the time series data by an increased correlation between the successive measurements of the functioning of the system (i.e., autocorrelation) [15]. While such dynamic indicators of resilience (DIORS) have been applied to study the resilience of, for example, human and ecological systems [14, 16, 17, 18], these have not yet been used to study the healthcare system resilience. Applying DIORS to time series data of healthcare system's functioning (continuity and quality of essential health services) could possibly be used to quantify the resilience of the healthcare system when faced with disruptive health events, such as the COVID‐19 pandemic. If these measures can be translated to real‐world monitoring of healthcare system resilience, they might help policymakers in anticipatory decision‐making and improve pandemic management [7].

### 1.3 Aim

The aim of this paper is two‐fold: First, to examine whether DIORs can be detected from time series data of the functioning of the Dutch healthcare system (i.e., the continuity and quality of essential health services, as measured with sick‐leave absenteeism rates among healthcare professionals) before, during, and after the COVID‐19 pandemic And second, to examine whether DIORs are indicative of the healthcare system's ability to withstand and respond to the shocks of the COVID‐19 pandemic (i.e., predictive validity of DIORs for healthcare resilience).

### 1.4 Hypothesis

We hypothesize to see an increased loss of resilience as the pandemic progressed, and we expect that low resilience is associated with a higher increase in sick‐leave, as each consecutive COVID‐19 wave infected more and more healthcare professionals and, thus, further depleted the resources, and thus, reducing its ability to maintain continuity and quality of essential health services. Furthermore, as different healthcare sectors of the Dutch healthcare system were affected differently by the pandemic, we expect to see heterogeneity in the loss of resilience between healthcare sectors, expecting a larger loss of resilience for healthcare sectors directly involved in COVID‐19 care (e.g., hospital and special care) compared to sectors less involved in COVID‐19 care (e.g., mental healthcare). Finally, due to regional differences in the impact of the pandemic within the Netherlands, we also expect to see regional heterogeneity in the loss of resilience between regions, expecting a larger loss of resilience for regions most affected by the pandemic (e.g., regions in the southern part of the Netherlands) compared to regions less affected by the pandemic (e.g., regions in the northern part the Netherlands; see [19] for an overview and description of the Dutch safety regions).

## 2 Methods

### 2.1 Study Design

This study employed a retrospective observational design, combining expert‐informed indicator selection through a structured questionnaire with secondary time‐series analysis of sick‐leave data to assess healthcare system functioning during the COVID‐19 pandemic, to analyse resilience using DIORs.

### 2.2 Data

#### 2.2.1 Functioning Healthcare System

DIORs are estimated by examining the recovery from natural occurring stressors, reflected in time series by changes in the fluctuations of the functioning of a system [12, 13, 14]. To apply DIORs to examine the resilience of the Dutch healthcare system to the COVID‐19 pandemic we therefore needed time series of the Dutch healthcare system's functioning before, during and after the pandemic. Healthcare system functioning was operationalized as the ability of the healthcare system to maintain continuity and quality of essential health services (i.e., healthcare availability). It is important to note that while healthcare system functioning can be assessed through various dimensions, including healthcare availability [20], cost‐effectiveness [21], and overall health outcomes [22], this study focuses on healthcare availability as a proxy for healthcare system functioning. This choice was motivated by the fact that the availability of healthcare services was one of the most extensively monitored aspects of healthcare performance to assess the strain on the Dutch healthcare system during the COVID‐19 pandemic. National monitoring efforts, such as the COVID‐19 dashboard developed by the Ministry of Health and reports by the Dutch Healthcare Authority, primarily focused on indicators related to hospital capacities and ICU capacity, reflecting a strong emphasis on system availability [23, 24]. Healthcare availability can be measured using various indicators (e.g., available ICU beds, waiting time for healthcare services), to select the most relevant indicator of healthcare availability, 45 healthcare experts (primary, acute, chronic and psychiatric care) completed a questionnaire in which they selected the five most relevant indicators of healthcare availability from a total of 25 indicators of healthcare availability used by the Dutch Healthcare Authority to asses the healthcare availability during the COVID‐19 pandemic [24]. Of the 25 indicators evaluated (see Table S14), several (e.g., *pressure on follow‐up care* and *waiting times)* were rated highly by experts but could not be included due to the lack of suitable publicly available datasets. *Sick‐leave among healthcare professionals* (i.e., available healthcare workers) was both highly rated and supported by comprehensive publicly available data, making it a feasible and meaningful measure of healthcare availability, and thus for healthcare system functioning, in this study.

#### 2.2.2 Sick‐Leave Absenteeism Among Healthcare Professionals

Data on sick‐leave absenteeism among healthcare professionals in the Netherlands came from the research agency Vernet [25]. Data included the monthly percentage of healthcare professionals that is on *short‐term sick‐leave* (shorter than 90 days) and *long‐term sick‐leave* (longer than 90 days) between January 1 2019, and December 31 2022. These data were specified for each healthcare sector in the Netherlands (mental healthcare (MHC), disabled care (DC), nursing and home care (NHC), and hospital and special care (HSC)) and in in all 25 safety regions in the Netherlands. Monthly sick‐leave scores ranged from 0 (0% healthcare professionals employed are on sick‐leave in a specific month) to 100 (100% healthcare professionals are on sick‐leave in a specific month).

#### 2.2.3 COVID‐19 Waves

The COVID‐19 waves in the Netherlands were used to specify the period in which the healthcare systems was put under pressure and we expected a decline in resilience. The beginning and end of each COVID‐19 wave was determined by the dates mentioned in the government reports of Statistics Netherlands (CBS) on excess mortality due to the COVID‐19 pandemic [26, 27]. Three COVID‐19 waves were distinguished; the *first wave* (March 1, 2020 – May 10, 2020), the *second wave* (September 21, 2020 – May 31, 2021) and the *third wave* (August 16, 2021 − December 21, 2021). The time period before the first wave was labelled as *pre‐pandemic* (January 1, 2019 – February 29, 2020) and the time period after the third wave was labelled as *post‐pandemic* (December 22, 2021 − December 1, 2022).

### 2.3 Analysis

From the sick leave time series we estimated two metrics of DIORs as measures of resilience of the Dutch Healthcare system: increase of *lag‐1 autocorrelation* (ACF1) and increase of *standard deviation* (SD) [28, 29]. Previous research suggests that increases both DIORs are valid measures of the resilience in other complex systems [16, 18, 30]. The goal of these DIORs was to examine changes in the ‘recovery time’ (ACF1) and variability (SD) in the functioning of the healthcare system (i.e., indicated by the changes in sick‐leave rates) before, during, and after the pandemic. Moving window techniques were used to estimate the ACF1 and SD over time. All analyses were performed on the short‐term and long‐term sick‐leave data, for each healthcare sector, for each safety region the Netherlands, and for all healthcare sectors and all safety regions combined.

#### 2.3.1 Change in Sick‐Leave After the Pandemic

Change scores were calculated by subtracting the average sick‐leave rate during the pre‐pandemic period from the average sick‐leave rates during the post‐pandemic period. To test whether sick‐leave rates increased significantly after the pandemic, Mann Whitney U tests were performed on the average post‐pandemic sick‐leave rates compared to average pre‐pandemic sick‐leave rates. We expected higher sick‐leave rates at post‐pandemic compared to pre‐pandemic.

#### 2.3.2 Estimate DIORs

##### 2.3.2.1 Detrending

Non‐stationarities in the mean of a time‐series can lead to spurious indications of reduced resilience [31]. Previous research has shown that Gaussian detrending performs well in detrending when nonlinear changes are present in the data [32, 33]. Before estimating DIORs, we therefore detrended the time‐series using simple Gaussian detrending with bandwidths [3, 4, 5, …, 20]. See Supporting materials for in‐dept description of detrending method.

##### 2.3.2.2 Estimate DIORs: Moving Window Technique

*Autocorrelation* was calculated with the ACF1, which indicates whether current monthly sick‐leave rates (at any given month in the time series) depends on the sick‐leave rates of the previous month. A high ACF1 indicates that monthly sick‐leave rates are strongly related to the sick‐leave rates of the previous month, indicative of slow recovery, which in turn is indicative of low resilience. A low ACF1 indicates that monthly sick‐leave rates are not (or weakly) related to the sick‐leave rates of the previous month, indicative of fast recovery, which in turn is indicative of high resilience [29, 34].

V*ariability* was calculated with the standard deviation (SD), which indicates the variability in sick‐leave rates. A high SD indicates strong fluctuations in monthly sick‐leave rates over time, which is indicative of low resilience; a low SD indicates small fluctuations in monthly sick‐leave rates over time, which is indicative of high resilience [28, 29].

To estimate the patterns of change in ACF1 and SD before, during and after the pandemic, moving window techniques were used on the detrended time series. A backward rolling window uses data from the past to estimate the current value of the ACF1 and SD at each point in time. The choice of rolling window length is a trade‐off between data availability and reliability of estimation. Since we are interested in the changes in ACF1 and SD during the pandemic, moving window techniques were applied with rolling window sizes of at least 27% of the length of time series data (i.e., the 13 months before the pandemic). As such, monthly ACF1 and SD scores were estimated using the data from the previous 13 months (February 2020 to December 2022). We chose this rolling window size since we are interested in changes in DIORs from the beginning of the pandemic onwards, a larger window size would result in missing DIORs estimates for the first months of the pandemic.

##### 2.3.2.3 Examine Change in DIORs During the Pandemic

We first visually inspected the ACF1 and SD estimates on whether resilience decreased following each COVID‐19 wave (and whether it recovered once the wave subsided), expecting a rise in DIORs during COVID‐19 waves and a decline in DIORs after the waves subsided. To illustrate the impact of the bandwidth choice for Gaussian detrending we plotted the DIORs estimates for the narrowest bandwidth (3), the widest bandwidth (20) and for a bandwidth somewhere in‐between narrow and wide (7).

To examine whether resilience declined during the pandemic, we calculated the Kendall's τ correlations between the ACF1 and SD estimates and their time index during the pandemic. Kendall's τ coefficient is the most popular statistic to quantify trends of DIORs [12, 32, 35, 36] as they are suited for capturing nonlinear monotonic associations typically seen in DIORs [37, 38]. A positive Kendall's τ indicates an increase in ACF1 and SD over time; that is, a loss of resilience during the pandemic. A negative Kendall's τ indicates an increase in ACF1 and SD over time; that is, an increase in resilience during the pandemic. We expected a positive Kendall's τ coefficient for both the ACF1 and SD during the pandemic.

##### 2.3.2.4 Compare DIORs Healthcare Sectors

To test our hypotheses that healthcare sectors most involved in the pandemic showed a greater loss in resilience compared to the healthcare sectors least involved in the pandemic, we correlated (Pearson) the Kendall's τ statistics of ACF1 and SD of each healthcare sector with its change in sick‐leave rates after the pandemic. Expecting a positive correlation between the loss in resilience (positive Kendall's τ) and the increase in sick‐leave rates among healthcare workers during the pandemic. More specifically, we expected a higher increase in sick‐leave rates in sectors that showed a greater loss of resilience during the pandemic (higher Kendall's τ) compared to sectors that showed a smaller loss (or increase) of resilience.

##### 2.3.2.5 Compare DIORs Safety Regions

To examine whether safety regions most affected by the pandemic showed a greater loss of resilience compared to less affected regions, we correlated (Pearson's *r*) the Kendall's τ statistics of ACF1 and SD over time with the region's change in sick‐leave rates after the pandemic. Expecting a positive correlation between loss in resilience (positive Kendall's τ) and the increase in sick‐leave rates during the pandemic. More specifically, we expected a higher increase in sick‐leave absenteeism for regions that showed a greater loss of resilience during the pandemic (higher Kendall's τ) compared to regions that showed a smaller loss of resilience.

#### 2.3.3 Sensitivity Analysis

Since window size and detrending both affect the moving window estimates, we conducted sensitivity analysis to improve the robustness of the results. To examine the *robustness* of the predetermined rolling window size of 27% (i.e., first 13 months before the pandemic), we compared the DIORs estimate with window sizes [15%, 16%, …, 50% of the data] and bandwidths [3, 6, 7, …, 75] on the national data on short‐ and long‐term sick‐leave rates. To improve the robustness of our results we also conducted *sensitivity analyses* on bandwidths [3, 4, 5, …, 20] for Gaussian detrending on all analysis (e.g., Kendall's τ and Pearson's *r*). See Supporting materials for in‐dept description of the robustness & sensitivity analysis.

#### 2.3.4 Software

All analysis were performed in in R v.4.2.2 [39]. Gaussian detrending was performed using R‐package ‘stats’ [39]. Estimations of the ACF1 and SD over time using the moving window technique were performed using R‐package ‘earlywarnings’ [40].

### 2.4 Ethics Approval and Consent to Participate

This study utilized anonymized collected data and did not involve interventions or the processing of personally identifiable information. Consequently ethical approval was not required. The expert consultation conducted for indicator selection adhered to institutional ethical guidelines.

## 3 Results

### 3.1 Aim 1: Detecting DIORs From Time Series Data

#### 3.1.1 Short‐Term Sick‐Leave in the Netherlands

**Figure 1:**
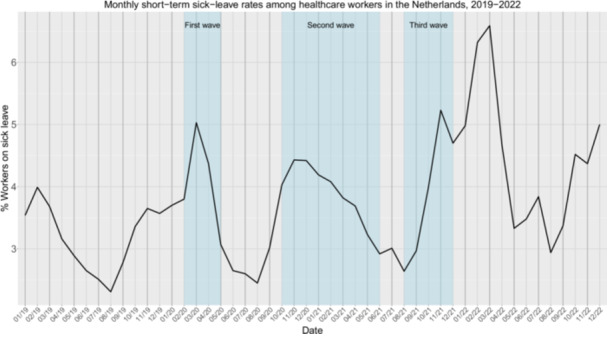
Monthly short‐term sick‐leave rates (% sick‐leave absenteeism among healthcare that takes less than 90 days) among healthcare workers in the Netherlands from January 2019 to December 2022. The blue vertical bars indicate the first, second, and third COVID‐19 waves in the Netherlands.

We found significantly higher short‐term sick‐leave rates after the pandemic (*M* = 4.5%) compared to before the pandemic (*M* = 3.2%), *W* = 28, *p* < 0.001 (Table S1). Figure 1 demonstrates an increase in sick leave rates with each COVID‐19 wave, resulting in an accumulating increase in short‐term sick‐leave absenteeism rates over time. Finally, sick leave rates continued to remain higher than before the pandemic once the pandemic subsided.

##### 3.1.1.1 DIORs

For ACF1 we found significantly positive Kendall's τ statistics during the pandemic across all bandwidth values (*M* τ = 0.46; range = 0.41–0.49; Table S2). This indicates an increased recovery time in sick leave absenteeism rates as the pandemic progresses. SD did not show significant (positive) change during the pandemic across any bandwidth values (*M* τ = 0.49; range = −0.30–0.67; Table S2).

We illustrate the DIORs estimates after Gaussian detrending with bandwidths 3, 7 and 20 in Figure S1. During the first and second COVID‐waves we see an increase in ACF1, indicating that it took increasingly more time for the healthcare system to recover from higher sick‐leave rates. After the second COVID‐wave we see a decline in ACF1 scores, indicating a reduction in recovery time from higher sick‐leave rates. Although SD did not increase significantly during whole the pandemic, the first half of the pandemic showed an overall increase in variability, followed by a decline during the second half of the pandemic.

#### 3.1.2 Long‐Term Sick‐Leave in the Netherlands

**Figure 2:**
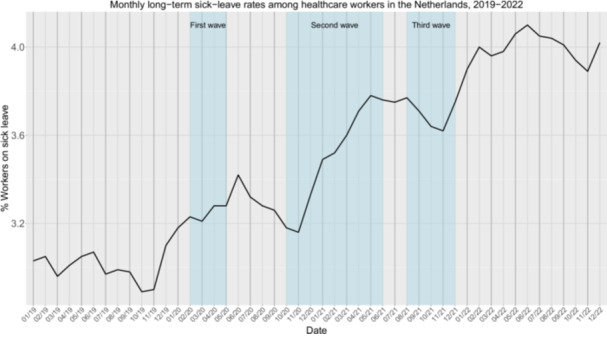
Monthly long‐term sick‐leave rates (% sick‐leave absenteeism among healthcare that takes more than 90 days) among healthcare workers in the Netherlands from January 2019 to December 2022. The blue vertical bars indicates the first, second, and third COVID‐19 waves in the Netherlands.

We found significantly higher long‐term sick‐leave rates after the pandemic (*M* = 4%) compared to before the pandemic (*M* = 3%), *W* = 0, *p* < 0.001 (Table S1). Long‐term sick‐leave rates in Figure 2 demonstrate a rise in sick leave rates after each COVID‐19 wave, resulting in an accumulating increase in long‐term sick‐leave rates during the pandemic. Furthermore, we see that long‐term sick‐leave rates continues to increase after the pandemic subsided, with no signs of recovery as of December 2022.

##### 3.1.2.1 Diors

For ACF1 we found significant positive Kendall's τ statistics during the pandemic across all bandwidth values (*M* τ = 0.52; range = 0.41–0.61; Table S3). For SD we found significant positive Kendall's τ statistics during the pandemic across most bandwidth values, except bandwidths 3,4,5 (*M* τ = 0.49; range = −0.30 –0.67; Table S3).

We illustrate the DIORs estimates for bandwidths 3, 7 and 20 in Figure S2. We see a (delayed) increase in ACF1 during the pandemic for all bandwidths,. For SD estimates we only see increase in variability for bandwidths 7 and 20.

#### 3.1.3 Short‐Term Sick‐Leave Among Healthcare Sectors in the Netherlands

**Figure 3:**
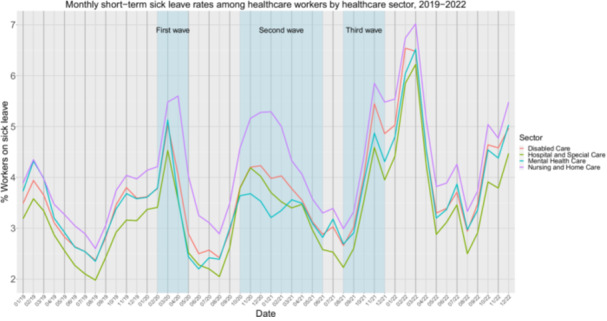
Monthly short‐term sick‐leave rates (% sick‐leave absenteeism among healthcare that takes less than 90 days) among healthcare workers for each healthcare sector in the Netherlands from January 2019 to December 2022. The three blue vertical bars indicate the time periods of the first, second and third COVID‐19 waves.

All healthcare sectors showed an increase in the short‐term sick‐leave rates during the pandemic (Table S4). Short term sick‐leave rates increased after each COVID‐wave (Figure 3), resulting in an accumulating increase in healthcare workers that is on sick‐leave during the pandemic.

##### 3.1.3.1 Diors

ACF1 increased significantly during the pandemic in healthcare sectors DC, NHC, HSC, indicating a loss of resilience. SDs did not increase in sectors DC, NHC, HSC (Table S5 and Figure S3). For example, while the NHC sector showed significant positive Kendall's τ statistics in SD across most bandwidth values (*M* τ = 0.33; range = −0.12–0.61), DC sector showed no significant Kendall's τ statistics across any bandwidth values (*M* τ = 0.05; range = −0.23– 0.16) and the MH sector showed significant *negative* Kendall's τ statistics across most bandwidth values (*M* τ = −0.35; range = −0.47– −0.15).

We illustrate the DIORs estimates for bandwidths 3, 7 and 20 for each healthcare sector in Figures S4 to S7. We see that ACF1 gradually increased during the pandemic and gradually declined once the pandemic subsided. This indicates an increased recovery rate as the during the pandemic and a shortening of recovery rate once the pandemic subsided. SD estimates showed inconsistent changes across the healthcare sectors.

#### 3.1.4 Long‐Term Sick‐Leave Among Healthcare Sectors in the Netherlands

**Figure 4:**
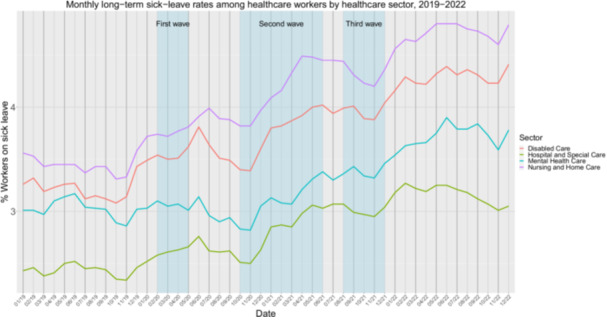
Monthly long‐term sick‐leave rates (% sick‐leave absenteeism among healthcare that takes more than 90 days) among healthcare workers for each healthcare sector in the Netherlands from January 2019 to December 2022. The three blue vertical bars indicate the time periods of the first, second and third COVID‐19 waves.

All healthcare sectors showed an increase in the long‐term sick‐leave rates during the pandemic (Table S7). Looking at Figure 4 we see that healthcare sectors differed in their absolute sick‐leave rate; with the HSC sector consistently having the lowest long‐term sick‐leave rates and NHM consistently having the highest sick‐leave rates. While the impact of the COVID‐19‐waves on sick‐leave rates was not as pronounced as in the short‐term sick‐leave rates, there seem to be a temporary rise in long‐term sick‐leave a few months after each COVID‐wave (to be expected as sick‐leave count as long‐term sick‐leave after 90 days).

##### 3.1.4.1 DIORs

We found robust evidence for significant increase in ACF1 for the NHC sector (*M* τ = 0.60; range = 0.46– 0.72; Figure S8 and Table S8). For the SD estimates we found robust evidence for significant increase in variability for the HSC sector (*M* τ = 0.68; range = 0.53–0.75; Figure S8 and Table S8).

We illustrate the DIORs estimates for bandwidths 3, 7 and 20 for each healthcare sector in Figures S9 to S12. ACF1 estimates showed a rise during the pandemic and decline once the pandemic subsided for bandwidth 3,. SD estimates in sectors HSC and MH showed a rise during the pandemic and decline once the pandemic subsided for bandwidths 3,7 and 20.

#### 3.1.5 Short‐Term Sick‐Leave Among Dutch Safety Regions

**Figure 5:**
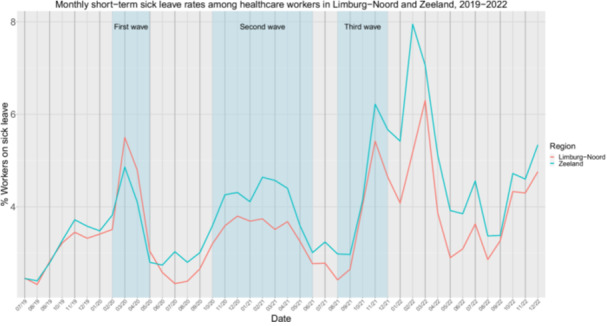
Monthly short‐term sick‐leave rates (% sick‐leave absenteeism among healthcare that takes less than 90 days) among healthcare workers in safety regions Limburg‐Noord and Zeeland from July 2019 to December 2022. The three blue vertical bars indicate the time periods of the first, second and third COVID‐19 waves.

Short‐term sick‐leave rates increased in all safety regions after the pandemic, with heterogeneity in the rate of change during the pandemic (Table S10). To illustrate we plotted short‐term sick‐leave rates for the region with the highest increase in sick‐leave (Zeeland; *M*_*change*_ = 1.84%, *W* = 6, *p* < 0.001) and lowest increase in sick‐leave (Limburg‐Noord; *M*_*change*_ = 1.05%, *W* = 15, *p* = 0.02) (Figure 5). Interestingly, Zeeland had lower sick‐leave rates compared to Limburg‐Noord during the first wave, however, following the first wave Zeeland had consistently higher sick‐leave rates.

##### 3.1.5.1 DIORs

We found robust increases in ACF1 across most (20 of the 25) safety regions, indicating a loss of resilience (i.e., increased recovery time) during the pandemic (Figure S13). However, we only found robust evidence for an increase in SD for regions Friesland (M τ = 0.63; range = −0.25– 0.82), Zeeland (M τ = 0.23; range = −0.13– 0.38), and Zuid‐Holland‐Zuid (M τ = 0.18; range = −0.03– 0.37), all other regions showed an decrease in SD during the pandemic.

We also plotted the DIORs estimates of Limburg‐Noord (Figure S14) and Zeeland (Figure S15) for bandwidths 3, 7 and 20. Figure for Zeeland did not show changes in ACF1 over time, however showed an gradual increase in SD over time (Figure S14). For Limburg‐Noord we see an increase in ACF1 (slower recovery time) during the first half of the pandemic and a decrease in ACF1 (faster recovery time) during the second half of the pandemic. However SDs showed the opposite; a decline during the first half of the pandemic and an increase during the second half of the pandemic.

#### 3.1.6 Long‐Term Sick‐Leave Among Dutch Safety Regions

All safety regions increased in long‐term sick‐leave after the pandemic compared to before the pandemic (Table S12). To illustrate this we plotted the monthly percentage of healthcare workers on long‐term sick‐leave for safety region with the highest increase in sick‐leave after the pandemic (Zeeland; *M*_*change*_ = 1.74%, *W* = 0, *p* < 0.001) and the safety region with the lowest increase in sick‐leave after the pandemic (Limburg‐Noord; *M*_*change*_ = 0.44%, *W* = 0, *p* < 0.001) in Figure S16. Interestingly, during the first COVID‐19 wave Zeeland had similar sick leave rates as Limburg. However, during the second COVID‐19 wave, long‐term sick‐leave increased rapidly in Zeeland while it remained stable in Limburg‐Noord.

##### 3.1.6.1 DIORs

We found increases in ACF1 across most safety regions, indicating a loss of resilience (i.e., increased recovery time) during the pandemic (Figure S17). It should be noted however that five regions showed a decline in ACF1 over time, indicating an increase in resilience during the pandemic. Kendall's τ of the SDs did not show evidence for an increase in variability across safety regions. Seven regions (e.g., Friesland, Groningen, and IJselland) showed an increase in SD over time across all bandwidths, while six regions (e.g., Brabant‐Noord and Hollands‐Midden) showed a decline in SD over time across all bandwidth.

To illustrate we plotted the DIORs estimates of Limburg‐Noord (Figure S18) and Zeeland (Figure S19) for bandwidths 3, 7 and 20. For Limburg‐Noord we see inconsistent patterns in ACF1 and SD across detrending method. For Zeeland we see a clear increase in both ACF1 and SD during the pandemic. This indicates a decline of resilience, as indicated with an increased recovery time and variability in long‐term sick leave rates.

### 3.2 Aim 2: Predictive Validity of DIORs of Healthcare Resilience

To examine the predictive validity of DIORs for healthcare resilience we correlated the change in DIORs with the change in sick‐leave rates after the pandemic across *healthcare sectors* and across *safety regions*. DIORs have predictive validity if they show a significant positive association with long term sick leave.

#### 3.2.1 Healthcare Sectors

##### 3.2.1.1 Association DIORs and Short‐Term Sick‐Leave Absenteeism

No relationship was found between the change in DIORs during the pandemic and the change in short‐term sick‐leave rates after the pandemic across healthcare sectors (Table S6).

##### 3.2.1.2 Association DIORs and Long‐Term Sick‐Leave Absenteeism

Shorter bandwidths (3, 5–11) showed a positive significant relationship between the change in ACF1 during the pandemic and the change in long‐term sick‐leave rates after the pandemic (see Table S9 for the statistics). For SD we did not find a relationships between its change during the pandemic and the change in sick‐leave during the period after the pandemic.

#### 3.2.2 Safety Regions

##### 3.2.2.1 Association DIORs and Short‐Term Sick‐Leave Absenteeism

No relationship was found between the change in ACF1 during the pandemic and the change in short‐term sick‐leave rates after the pandemic across safety regions (Table S11). However, there was a positive association between the change in SD and the change in short‐term sick‐leave rates after the pandemic across safety regions.

##### 3.2.2.2 Association DIORs and Long‐Term Sick‐Leave Absenteeism

Across safety regions, there was no significant relationship between the change in ACF1 and SD during the pandemic and the change in long‐term sick‐leave rates after the pandemic (Table S13).

## 4 Conclusion

In this first study on the applicability and validity of DIORs to examine healthcare system resilience, we found tentative evidence for using DIORs to examine the Dutch healthcare system's resilience to the shocks of the COVID‐19 pandemic. As expected, we found that the estimated DIORs increased during the pandemic, indicated a loss of resilience of the Dutch healthcare system during the COVID‐19 pandemic.

Comparing the DIORs across healthcare sectors, we found evidence for a loss of resilience during the pandemic, with a greater loss of resilience in the healthcare sectors directly involved in COVID‐19 care (e.g., HSC) compared to sectors not directly involved in COVID‐19 care (e.g., MH). Looking at the relationship between changes in DIORs and changes in sick leave among healthcare sectors, we found tentative evidence that a loss of resilience is predictive of a decline in sick‐leave rates after the pandemic.

Comparing the DIORs across safety regions in the Netherlands we did not find consistent and robust evidence for loss of resilience during the pandemic, nor did we find consistent evidence that points to a greater loss of resilience for the regions that were more heavily affected by the pandemic. Contrary to our expectations we found that the region most affected by the pandemic (Limburg‐Noord) showed a smaller decline in resilience during the pandemic compared to the region least affected by the pandemic (Zeeland). A possible explanation might be an increased adaptive capacity of Limburg‐Noord due to the high pressure during first COVID‐19 wave, making its healthcare workers, local authorities and policymakers better prepared for the second and third waves compared to Zeeland, which was less affected by the first wave and therefore might be less prepared for the impact of second (and third) wave. However, it should be noted that looking at all regions together, we did not find an association between changes in DIORs and the changes in sick‐leave rates.

### 4.1 Limitations

Firstly, since we do not know the exact mechanism underlying the functioning of the healthcare system (i.e., interactions and feedback loops most important for changing the overall functioning of the healthcare system), it therefore remains unknown whether DIORs estimates from sick leave absenteeism rates can be generalised to the other measures of functioning as well (e.g., available ICU beds, waiting time to healthcare services). Secondly, due to the lack of historical data on sick leave rates from the period before the COVID‐19 pandemic, we were not able to examine whether the trend in DIORs during the pandemic were significantly different from the period before the pandemic. Thirdly, during the COVID‐19 pandemic healthcare workers from the less impacted regions in the Netherlands were moved to the more heavily impacted regions to alleviate the pressure in that regions. This could have altered the proportions of sick leave rates during the course of the pandemic, which in turn could have affected our DIORs estimates and its relationship with the COVID‐19 pandemic. Fourthly, sick‐leave data was only measured on a monthly basis, thereby we missed the weekly/daily dynamics in healthcare functioning over time. Finally, since we tested multiple hypotheses simultaneously, there is a higher risk of making a Type I error due to the random chance in hypotheses testing.

### 4.2 Future Directions and Implications

The use of DIORs for examining healthcare system resilience should be replicated on other time series of healthcare functioning (e.g. available ICU bed, waiting time for surgery etc.) and across other time scales (both shorter and longer than studied here) with a larger database of historical data (e.g., a years before the COVID‐19 pandemic). Secondly, to get a more complete picture of healthcare system resilience it is important to replicate these findings during other disruptive health events (e.g., natural disasters or other types of pandemics). Thirdly, healthcare systems do not exist in isolation and other systems (e.g., the economy or educational) also influence the healthcare system in their unique way [41]. To better understand the resilience of the healthcare system it would therefore be important to take into account the interactions with other systems. Fourthly, because the Netherlands has a universal healthcare system [42], we only examined DIORs in a public healthcare setting. Previous research showed that private and public healthcare systems responded differently to the COVID‐19 pandemic [43, 44]. Future research should examine whether our results can be replicated in a private healthcare setting. Fifthly, it should be noted that there is a relationship between policy measures and the availability of healthcare workers; i.e., mandatory testing protocols temporary decreases the number of available staff as positively tested individuals are bound to stay home. While such policy measures prevent further transmission to colleagues, they also increase the workload for the remaining healthcare workers increases, possibly increasing sick‐leave rates on the long‐term. Future research should examine the impact of such policy measures on sick‐leave rates and how it affects the estimation of DIORs. Finally, to address the multiple comparisons problem mentioned in the limitations, future research should quantify fewer, and more specific hypotheses regarding the validity of using DIORs. For example, by testing whether sudden declines in functioning of the healthcare system can be predicted by prior increases in DIORs.

This study has several implications for the use of DIORs to examine the healthcare resilience. First, it supports the idea that healthcare systems can be understood as complex dynamical systems, which allows new methods and generates new hypotheses regarding the resilience of healthcare systems [11]. Previous attempts to measure healthcare system resilience have so far mostly remained theoretical and/or do not take into account the interconnected nature of healthcare systems [8]. Our approach allowed to measure healthcare resilience directly by examining patterns in healthcare functioning that arise from these complex interactions that make up the healthcare system. Furthermore, DIORs estimated from real‐time time‐series of healthcare functioning may be used as a monitoring value for policy‐making to better prepare for future disruptions in healthcare. For example as an ‘early warning’ informing policymakers when they need to adapt/change current measures/strategies.

## 5 Conclusion

Our results indicate that DIORs, estimated from time series data of sick‐leave rates among healthcare professionals, potentially provide useful insights into the healthcare systems ability to provide quality healthcare services in the face of disruptive health events, such as pandemics. This preliminary study illustrates the value of DIORs as measures of resilience of the Dutch healthcare system before, during and after a pandemic. Future research should replicate these findings and extend validity testing of DIORs to other aspects of healthcare systems. Policy makers could experiment in applying such resilience measures in better prepare for future disruptions and understanding of healthcare functioning.

## Author Contributions

**Tom H Oreel:** conceptualization, formal analysis, investigation, writing – original draft, writing – review and editing, methodology. **Sophie Hadjisotiriou:** Investigation, writing – review and editing. **Vítor V Vasconcelos:** conceptualization, formal analysis, methodology, writing – review and editing. **Vincent A W J Marchau:** writing – review and editing, funding acquisition. **Etiënne AJA Rouwette:** writing – review and editing, funding acquisition. **Rick Quax:** methodology, conceptualization, writing – review and editing, funding acquisition. **Vittorio Nespeca:** writing – review and editing. jannie coenen: writing – review and editing. **Hubert P L M Korzilius:** writing – review and editing, funding acquisition. **Heiman Wertheim:** writing – review and editing, funding acquisition. **Marcel G M Olde Rikkert:** conceptualization, investigation, methodology, writing – review and editing, funding acquisition.

## Ethics Statement

All authors have read and approved the final version of the manuscript Tom Oreel had full access to all of the data in this study and takes complete responsibility for the integrity of the data and the accuracy of the data analysis.

## Conflicts of Interest

The authors declare no conflicts of interest.

## 1 Transparency statement

The lead author Tom Oreel affirms that this manuscript is an honest, accurate, and transparent account of the study being reported; that no important aspects of the study have been omitted; and that any discrepancies from the study as planned (and, if relevant, registered) have been explained.

## Supporting information

Supplementary File.

## Data Availability

The data that support the findings of this study are available in Vernet at https://inzetzorg.arbeidsmarktinbeeld.nl/inzetzorg-landelijk-dashboard?sectionId=5112. These data were derived from the following resources available in the public domain: ‐ Vernet 2022, https://inzetzorg.arbeidsmarktinbeeld.nl/inzetzorg-landelijk-dashboard?sectionId=5112
