# Supplementary material for: Measuring Health System Resilience During the COVID‐19 Pandemic Using Dynamic Indicators of Resilience Based on Sick‐Leave Data
Source: Health Sci Rep. 2025 Jun 23;8(6):e70789. doi: 10.1002/hsr2.70789 (PMC12183390; doi:10.1002/hsr2.70789)
Supplement: Supplementary file 1 — Supplementary File. [file HSR2-8-e70789-s001.docx]

**Supplementary material**

**Table S1**: Average short- and long-term sick-leave rates at pre- and post-pandemic in the Netherlands

| **Sick-leave data** | **Period** | **Average sick leave rates** |
| --- | --- | --- |
|  |  |  |
| Short-term sick leave |  |  |
|  | pre-pandemic | 3.215 |
|  | post-pandemic | 4.452 |
|  | change | 1.237 (p=.005) |
| Long-term sick leave |  |  |
|  | pre-pandemic | 3.014 |
|  | post-pandemic | 3.996 |
|  | change | 0.982 (p<.001) |

* P values < .05 were considered significant

**Table S2**: Kendall’s τ of the DIORs from the short-term sick leave rates in the Netherlands.. The table displays the Kendall’s τ of the autocorrelation at lag-1 (ACF1) and standard deviation (SD) estimates for the short-term sick leave time series after linear detrending and Gaussian detrending (bandwidths 3-20). Kendall’s τ indicates the rate of change of the DIORs during the COVID-19 pandemic (March 2020 – December 2021). Significant Kendall’s τ are in bold.

| **Detrending** | **ACF1** | | **SD** | |
| --- | --- | --- | --- | --- |
|  | Kendall's τ | p-value | Kendall's τ | p-value |
| *Bandwidths* |  |  |  |  |
| 3 | **.407** | .006 | -.146 | .346 |
| 4 | **.423** | .004 | -.146 | .346 |
| 5 | **.423** | .004 | -.13 | .402 |
| 6 | **.439** | .003 | -.13 | .402 |
| 7 | **.455** | .002 | -.036 | .835 |
| 8 | **.486** | .001 | .020 | .917 |
| 9 | **.462** | .002 | .107 | .497 |
| 10 | **.486** | .001 | .162 | .294 |
| 11 | **.494** | .001 | .178 | .248 |
| 12 | **.470** | .001 | .225 | .140 |
| 13 | **.478** | .001 | .241 | .114 |
| 14 | **.486** | .001 | .249 | .102 |
| 15 | **.455** | .002 | .233 | .127 |
| 16 | **.462** | .002 | .225 | .140 |
| 17 | **.455** | .002 | .209 | .172 |
| 18 | **.455** | .002 | .194 | .207 |
| 19 | **.447** | .002 | .162 | .294 |
| 20 | **.447** | .002 | .162 | .294 |

* P values < .05 were considered significant

**Table S3**: Kendall’s τ of the DIORs from the long-term sick leave rates in the Netherlands. The table displays the Kendall’s τ of the autocorrelation at lag-1 (ACF1) and standard deviation (SD) estimates for the long-term sick leave time series after Gaussian detrending (bandwidths 3-20). Kendall’s τ indicates the rate of change of the DIORs during the COVID-19 pandemic (March 2020 – December 2021). Significant Kendall’s τ are in bold.

| **Detrending** | **ACF1** | | **SD** | |
| --- | --- | --- | --- | --- |
|  | Kendall's τ | p-value | Kendall's τ | p-value |
| *Bandwidths* |  |  |  |  |
| 3 | **.486** | .001 | **-.304** | .044 |
| 4 | **.565** | <.001 | -.107 | .497 |
| 5 | **.621** | <.001 | .178 | .248 |
| 6 | **.605** | <.001 | **.352** | .019 |
| 7 | **.581** | <.001 | **.447** | .002 |
| 8 | **.581** | <.001 | **.581** | <.001 |
| 9 | **.581** | <.001 | **.636** | <.001 |
| 10 | **.557** | <.001 | **.66** | <.001 |
| 11 | **.542** | <.001 | **.66** | <.001 |
| 12 | **.534** | <.001 | **.66** | <.001 |
| 13 | **.526** | <.001 | **.668** | <.001 |
| 14 | **.502** | .001 | **.668** | <.001 |
| 15 | **.494** | .001 | **.644** | <.001 |
| 16 | **.478** | .001 | **.636** | <.001 |
| 17 | **.47** | .001 | **.605** | <.001 |
| 18 | **.439** | .003 | **.605** | <.001 |
| 19 | **.431** | .004 | **.636** | <.001 |
| 20 | **.407** | .006 | **.652** | <.001 |

* P values < .05 were considered significant

**Table S4:** Average short-term sick-leave rates and corresponding DIORs from the short-term sick leave rates across healthcare sectors at pre- and post-pandemic.. P-values were determined with the Mann-Whitney U test.

| **Sector** | **Period** | **Average short-term sick leave rates** |
| --- | --- | --- |
|  |  |  |
| Mental Health Care |  |  |
|  | pre-pandemic | 3.291 |
|  | post-pandemic | 4.388 |
|  | change | 1.098 (*p* = .012) |
| Disabled Care |  |  |
|  | pre-pandemic | 3.22 |
|  | post-pandemic | 4.47 |
|  | change | 1.25 (*p* = .010) |
| Nursing and Home Care |  |  |
|  | pre-pandemic | 3.572 |
|  | post-pandemic | 4.899 |
|  | change | 1.327 (*p* = .007) |
| Hospital and Special Care |  |  |
|  | pre-pandemic | 2.84 |
|  | post-pandemic | 3.984 |
|  | change | 1.144 (*p* = .008) |

* P values < .05 were considered significant

**Table S5:** Kendall’s τ of the DIORs from the short-term sick leave rates across healthcare sectors. The table displays the Kendall’s τ of the autocorrelation at lag-1 (ACF1) and standard deviation (SD) estimates from the short-term sick leave time series Gaussian detrending (bandwidths 3-20). Kendall’s τ indicates the rate of change of the DIORs during the COVID-19 pandemic (March 2020 – December 2021). Significant Kendall’s τ are in bold.

| **Detrending** | **Mental Health Care** | | **Disabled Care** | | **Nursing & Home Care** | | **Hospital & Special Care** | |
| --- | --- | --- | --- | --- | --- | --- | --- | --- |
|  | ACF1 | SD | ACF1 | SD | ACF1 | SD | ACF1 | SD |
| *Bandwidths* |  |  |  |  |  |  |  |  |
| 3 | 0.217 | -0.186 | **0.391** | -0.233 | **0.344** | -0.115 | **0.486** | -0.11 |
| 4 | 0.281 | -0.178 | **0.47** | -0.186 | **0.462** | -0.083 | **0.47** | -0.115 |
| 5 | 0.257 | -0.154 | **0.486** | -0.13 | **0.542** | -0.036 | **0.455** | -0.13 |
| 6 | 0.186 | -0.17 | **0.494** | -0.083 | **0.605** | -0.036 | **0.447** | -0.036 |
| 7 | 0.178 | -0.257 | **0.526** | -0.02 | **0.605** | 0.107 | **0.407** | 0.012 |
| 8 | 0.186 | **-0.304** | **0.549** | 0.012 | **0.621** | 0.194 | **0.391** | 0.043 |
| 9 | 0.186 | **-0.368** | **0.557** | 0.067 | **0.597** | **0.265** | **0.423** | 0.115 |
| 10 | 0.17 | **-0.383** | **0.549** | 0.099 | **0.605** | **0.304** | **0.447** | 0.154 |
| 11 | 0.162 | **-0.415** | **0.534** | 0.138 | **0.613** | **0.375** | **0.455** | 0.202 |
| 12 | 0.154 | **-0.407** | **0.526** | 0.154 | **0.581** | **0.423** | **0.455** | 0.233 |
| 13 | 0.154 | **-0.407** | **0.518** | 0.162 | **0.581** | **0.518** | **0.439** | 0.257 |
| 14 | 0.138 | **-0.415** | **0.494** | 0.162 | **0.581** | **0.542** | **0.431** | 0.296 |
| 15 | 0.138 | **-0.415** | **0.494** | 0.146 | **0.565** | **0.557** | **0.431** | **0.304** |
| 16 | 0.115 | **-0.431** | **0.478** | 0.138 | **0.549** | **0.565** | **0.407** | **0.328** |
| 17 | 0.091 | **-0.439** | **0.47** | 0.138 | **0.542** | **0.597** | **0.383** | **0.328** |
| 18 | 0.083 | **-0.439** | **0.462** | 0.13 | **0.518** | **0.605** | **0.375** | **0.328** |
| 19 | 0.075 | **-0.447** | **0.455** | 0.107 | **0.502** | **0.597** | **0.368** | **0.328** |
| 20 | 0.02 | **-0.447** | **0.455** | 0.091 | **0.494** | **0.589** | **0.36** | **0.328** |

**Table S6**: Pearson correlations between Kendall’s τ of the DIORs and the change in short-term sick leave rates after the pandemic across healthcare sectors. The table displays the Pearson correlation coefficients and corresponding p-value of the Kendall’s τ of the autocorrelation at lag-1 (ACF1) and standard deviation (SD) estimates during the pandemic on the short-term sick leave data after Gaussian detrending (bandwidths 3-20). Significant correlations are indicated in bold.

| **Detrending** | **ACF1** | | **SD** | |
| --- | --- | --- | --- | --- |
|  | Pearson *r* change sick-leave | p-value | Pearson *r* change sick-leave | p-value |
| *Bandwidths* |  |  |  |  |
| 3 | 0.206 | 0.794 | 0.125 | 0.875 |
| 4 | 0.665 | 0.335 | 0.481 | 0.519 |
| 5 | 0.864 | 0.136 | 0.867 | 0.133 |
| 6 | 0.896 | 0.104 | 0.623 | 0.377 |
| 7 | 0.937 | 0.063 | 0.811 | 0.189 |
| 8 | **0.956** | 0.044 | 0.835 | 0.165 |
| 9 | 0.916 | 0.084 | 0.804 | 0.196 |
| 10 | 0.894 | 0.106 | 0.795 | 0.205 |
| 11 | 0.888 | 0.112 | 0.795 | 0.205 |
| 12 | 0.860 | 0.140 | 0.796 | 0.204 |
| 13 | 0.879 | 0.121 | 0.815 | 0.185 |
| 14 | 0.881 | 0.119 | 0.795 | 0.205 |
| 15 | 0.87 | 0.130 | 0.788 | 0.212 |
| 16 | 0.877 | 0.123 | 0.771 | 0.229 |
| 17 | 0.889 | 0.111 | 0.781 | 0.219 |
| 18 | 0.877 | 0.123 | 0.78 | 0.220 |
| 19 | 0.869 | 0.131 | 0.769 | 0.231 |
| 20 | 0.853 | 0.147 | 0.760 | 0.240 |

* P values < .05 were considered significant

**Table S7**: Average long-term sick-leave rates and corresponding DIORs from the long-term sick leave rates across healthcare sectors at pre- and post-pandemic. P-values were determined with the Mann-Whitney U test.

| **Sector** | **Period** | **Average Long-term sick leave rates** |
| --- | --- | --- |
|  |  |  |
| Mental Health Care |  |  |
|  | pre-pandemic | 3.022 |
|  | post-pandemic | 3.721 |
|  | change | 0.699 (p<.001) |
| Disabled Care |  |  |
|  | pre-pandemic | 3.235 |
|  | post-pandemic | 4.288 |
|  | change | 1.053 (p<.001) |
| Nursing and Home Care |  |  |
|  | pre-pandemic | 3.465 |
|  | post-pandemic | 4.708 |
|  | change | 1.244 (p<.001) |
| Hospital and Special Care |  |  |
|  | pre-pandemic | 2.44 |
|  | post-pandemic | 3.167 |
|  | change | 0.727 (p<.001) |

* P values < .05 were considered significant

**Table S8:** Kendall’s τ of the DIORs from the long-term sick leave rates across healthcare sectors. The table displays the Kendall’s τ of the autocorrelation at lag-1 (ACF1) and standard deviation (SD) estimates from the long-term sick leave time series after Gaussian detrending (bandwidths 3-20). Kendall’s τ indicates the rate of change of the DIORs during the COVID-19 pandemic (March 2020 – December 2021). Significant Kendall’s τ are in bold.

| **Detrending** | **Mental Health Care** | | **Disabled Care** | | **Nursing & Home Care** | | **Hospital & Special Care** | |
| --- | --- | --- | --- | --- | --- | --- | --- | --- |
|  | ACF1 | SD | ACF1 | SD | ACF1 | SD | ACF1 | SD |
| *Bandwidths* |  |  |  |  |  |  |  |  |
| 3 | **0.447** | **0.455** | **0.51** | **-0.375** | **0.455** | -0.399 | 0.154 | **0.534** |
| 4 | **0.336** | **0.51** | **0.47** | -0.281 | **0.715** | -0.265 | 0.083 | **0.565** |
| 5 | 0.162 | **0.47** | **0.423** | -0.138 | **0.7** | -0.036 | 0.004 | **0.589** |
| 6 | 0.004 | **0.344** | **0.399** | 0.004 | **0.692** | 0.281 | -0.178 | **0.605** |
| 7 | -0.059 | 0.265 | **0.344** | **0.012** | **0.676** | 0.502 | -0.099 | **0.613** |
| 8 | -0.091 | 0.194 | 0.336 | **0.051** | **0.66** | 0.613 | -0.115 | **0.652** |
| 9 | -0.075 | 0.162 | 0.304 | **0.051** | **0.628** | 0.676 | -0.036 | **0.692** |
| 10 | -0.091 | 0.146 | 0.257 | **0.067** | **0.628** | 0.684 | -0.004 | **0.715** |
| 11 | -0.067 | 0.162 | 0.233 | **0.059** | **0.621** | 0.684 | 0.02 | **0.708** |
| 12 | -0.020 | 0.178 | 0.202 | **0.043** | **0.613** | 0.684 | 0.043 | **0.715** |
| 13 | 0.028 | 0.178 | 0.130 | **0.043** | **0.597** | 0.692 | 0.091 | **0.715** |
| 14 | 0.020 | 0.162 | 0.115 | **0.012** | **0.573** | 0.644 | 0.091 | **0.708** |
| 15 | 0.059 | 0.194 | 0.083 | **-0.028** | **0.549** | 0.613 | 0.099 | **0.715** |
| 16 | 0.091 | 0.209 | 0.075 | **-0.083** | **0.542** | 0.573 | 0.091 | **0.715** |
| 17 | 0.115 | 0.225 | 0.043 | **-0.138** | **0.534** | 0.565 | 0.099 | **0.723** |
| 18 | 0.123 | 0.249 | 0.043 | **-0.194** | **0.542** | 0.557 | 0.083 | **0.731** |
| 19 | 0.123 | 0.241 | 0.02 | **-0.249** | **0.494** | 0.549 | 0.083 | **0.747** |
| 20 | 0.123 | 0.249 | 0.012 | **-0.273** | **0.486** | 0.534 | 0.091 | **0.747** |

**Table S9***:* Pearson correlations between Kendall’s τ of the DIORs and the change in long-term sick leave rates across healthcare sectors. among the Dutch healthcare sectors. The table displays the Pearson correlation coefficients and corresponding p-value of the Kendall’s τ of the autocorrelation at lag-1 (ACF1) and standard deviation (SD) estimates during the pandemic on the long-term sick leave data after Gaussian detrending (bandwidths 3-20*.* Significant correlations are indicated in bold.

| **Detrending** | **ACF1** | | **SD** | |
| --- | --- | --- | --- | --- |
|  | Pearson *r* change sick-leave | p-value | Pearson *r* change sick-leave | p-value |
| *Bandwidths* |  |  |  |  |
| 3 | **0.550** | 0.45 | **-0.955** | 0.045 |
| 4 | 0.895 | 0.105 | -0.947 | 0.053 |
| 5 | **0.964** | 0.036 | -0.899 | 0.101 |
| 6 | **0.972** | 0.028 | -0.587 | 0.413 |
| 7 | **0.993** | 0.007 | -0.131 | 0.869 |
| 8 | **0.996** | 0.004 | 0.086 | 0.914 |
| 9 | **0.994** | 0.006 | 0.148 | 0.852 |
| 10 | **0.980** | 0.02 | 0.158 | 0.842 |
| 11 | **0.967** | 0.033 | 0.147 | 0.853 |
| 12 | 0.944 | 0.056 | 0.119 | 0.881 |
| 13 | 0.864 | 0.136 | 0.128 | 0.872 |
| 14 | 0.855 | 0.145 | 0.078 | 0.922 |
| 15 | 0.798 | 0.202 | -0.008 | 0.992 |
| 16 | 0.772 | 0.228 | -0.087 | 0.913 |
| 17 | 0.702 | 0.298 | -0.13 | 0.870 |
| 18 | 0.707 | 0.293 | -0.175 | 0.825 |
| 19 | 0.662 | 0.338 | -0.194 | 0.806 |
| 20 | 0.640 | 0.360 | -0.218 | 0.782 |

* P values < .05 were considered significant

**Table S10**: Average short-term sick-leave rates for each safety region at pre- and post-pandemic.

| **Safety region** | **Period** | **Average short-term sick leave** |
| --- | --- | --- |
|  |  |  |
| Amsterdam-Amstelland | pre-pandemic | 2.939 |
|  | post-pandemic | 4.268 |
|  | change | 1.33 |
| Brabant-Noord | pre-pandemic | 2.983 |
|  | post-pandemic | 4.088 |
|  | change | 1.105 |
| Brabant-Zuidoost | pre-pandemic | 3.009 |
|  | post-pandemic | 4.206 |
|  | change | 1.197 |
| Drenthe | pre-pandemic | 3.2 |
|  | post-pandemic | 4.588 |
|  | change | 1.388 |
| Flevoland | pre-pandemic | 3.09 |
|  | post-pandemic | 4.84 |
|  | change | 1.75 |
| Friesland | pre-pandemic | 2.96 |
|  | post-pandemic | 4.487 |
|  | change | 1.527 |
| Gelderland-Midden | pre-pandemic | 3.086 |
|  | post-pandemic | 4.517 |
|  | change | 1.431 |
| Gelderland-Zuid | pre-pandemic | 2.974 |
|  | post-pandemic | 4.315 |
|  | change | 1.341 |
| Gooi en vechtstreek | pre-pandemic | 3.133 |
|  | post-pandemic | 4.573 |
|  | change | 1.44 |
| Groningen | pre-pandemic | 3.054 |
|  | post-pandemic | 4.477 |
|  | change | 1.422 |
| Haaglanden | pre-pandemic | 3.411 |
|  | post-pandemic | 4.91 |
|  | change | 1.499 |
| Hollands-Midden | pre-pandemic | 3.187 |
|  | post-pandemic | 4.457 |
|  | change | 1.27 |
| IJsselland | pre-pandemic | 2.77 |
|  | post-pandemic | 4.143 |
|  | change | 1.373 |
| Kennemerland | pre-pandemic | 3.366 |
|  | post-pandemic | 4.592 |
|  | change | 1.226 |
| Limburg-Noord | pre-pandemic | 3 |
|  | post-pandemic | 4.048 |
|  | change | 1.048 |
| Limburg-Zuid | pre-pandemic | 3.341 |
|  | post-pandemic | 4.499 |
|  | change | 1.158 |
| Midden- en West-Brabant | pre-pandemic | 3.049 |
|  | post-pandemic | 4.237 |
|  | change | 1.189 |
| Noord- en Oost-Gelderland | pre-pandemic | 3.073 |
|  | post-pandemic | 4.3 |
|  | change | 1.227 |
| Noord-Holland-Noord | pre-pandemic | 3.063 |
|  | post-pandemic | 4.282 |
|  | change | 1.22 |
| Rotterdam-Rijnmond | pre-pandemic | 3.431 |
|  | post-pandemic | 4.909 |
|  | change | 1.478 |
| Twente | pre-pandemic | 2.989 |
|  | post-pandemic | 4.239 |
|  | change | 1.251 |
| Utrecht | pre-pandemic | 3.199 |
|  | post-pandemic | 4.593 |
|  | change | 1.394 |
| Zaanstreek-Waterland | pre-pandemic | 3.491 |
|  | post-pandemic | 4.708 |
|  | change | 1.217 |
| Zeeland | pre-pandemic | 3.099 |
|  | post-pandemic | 4.941 |
|  | change | 1.842 |
| Zuid-Holland-Zuid | pre-pandemic | 3.063 |
|  | post-pandemic | 4.553 |
|  | change | 1.49 |

**Table S11***:* Pearson correlations between Kendall’s τ of the DIORs and the change in short-term sick leave rates across safety regions. The table displays the Pearson correlation coefficients and corresponding p-value of the Kendall’s τ of the autocorrelation at lag-1 (ACF1) and standard deviation (SD) estimates during the pandemic on the short-term sick leave data after Gaussian detrending (bandwidths 3-20)*.* Significant correlations are indicated in bold.

| **Detrending** | **ACF1** | | **SD** | |
| --- | --- | --- | --- | --- |
|  | Pearson *r* change sick-leave | p-value | Pearson *r* change sick-leave | p-value |
| *Bandwidths* |  |  |  |  |
| 3 | -0.067 | 0.751 | **0.532** | 0.006 |
| 4 | -0.003 | 0.990 | **0.554** | 0.004 |
| 5 | 0.068 | 0.746 | **0.472** | 0.017 |
| 6 | 0.074 | 0.727 | **0.476** | 0.016 |
| 7 | 0.049 | 0.816 | **0.459** | 0.021 |
| 8 | 0.067 | 0.751 | **0.464** | 0.019 |
| 9 | 0.064 | 0.762 | **0.451** | 0.024 |
| 10 | 0.051 | 0.809 | **0.431** | 0.032 |
| 11 | 0.065 | 0.757 | **0.429** | 0.032 |
| 12 | 0.048 | 0.818 | **0.410** | 0.042 |
| 13 | 0.038 | 0.855 | **0.406** | 0.044 |
| 14 | 0.055 | 0.796 | **0.414** | 0.040 |
| 15 | 0.082 | 0.698 | **0.429** | 0.032 |
| 16 | 0.079 | 0.709 | **0.421** | 0.036 |
| 17 | 0.076 | 0.720 | **0.408** | 0.043 |
| 18 | 0.092 | 0.662 | **0.405** | 0.045 |
| 19 | 0.103 | 0.623 | **0.409** | 0.042 |
| 20 | 0.113 | 0.591 | **0.410** | 0.042 |

* P values < .05 were considered significant

**Table S12**: Average long-term sick-leave rates for each safety region at pre- and post-pandemic.

| **Safety region** | **Period** | **Average short-term sick leave** |
| --- | --- | --- |
|  |  |  |
| Amsterdam-Amstelland | pre-pandemic | 2.657 |
|  | post-pandemic | 3.35 |
|  | change | 0.693 |
| Brabant-Noord | pre-pandemic | 3.033 |
|  | post-pandemic | 3.588 |
|  | change | 0.555 |
| Brabant-Zuidoost | pre-pandemic | 2.767 |
|  | post-pandemic | 3.572 |
|  | change | 0.805 |
| Drenthe | pre-pandemic | 3.406 |
|  | post-pandemic | 4.596 |
|  | change | 1.19 |
| Flevoland | pre-pandemic | 2.773 |
|  | post-pandemic | 4.417 |
|  | change | 1.644 |
| Friesland | pre-pandemic | 3.267 |
|  | post-pandemic | 4.407 |
|  | change | 1.14 |
| Gelderland-Midden | pre-pandemic | 2.841 |
|  | post-pandemic | 3.995 |
|  | change | 1.154 |
| Gelderland-Zuid | pre-pandemic | 2.747 |
|  | post-pandemic | 3.727 |
|  | change | 0.98 |
| Gooi en vechtstreek | pre-pandemic | 2.934 |
|  | post-pandemic | 3.723 |
|  | change | 0.788 |
| Groningen | pre-pandemic | 3.43 |
|  | post-pandemic | 4.329 |
|  | change | 0.899 |
| Haaglanden | pre-pandemic | 3.204 |
|  | post-pandemic | 4.336 |
|  | change | 1.132 |
| Hollands-Midden | pre-pandemic | 2.686 |
|  | post-pandemic | 3.658 |
|  | change | 0.972 |
| IJsselland | pre-pandemic | 2.826 |
|  | post-pandemic | 3.724 |
|  | change | 0.898 |
| Kennemerland | pre-pandemic | 2.966 |
|  | post-pandemic | 3.757 |
|  | change | 0.791 |
| Limburg-Noord | pre-pandemic | 3.179 |
|  | post-pandemic | 3.619 |
|  | change | 0.441 |
| Limburg-Zuid | pre-pandemic | 3.413 |
|  | post-pandemic | 4.468 |
|  | change | 1.055 |
| Midden- en West-Brabant | pre-pandemic | 2.84 |
|  | post-pandemic | 3.868 |
|  | change | 1.028 |
| Noord- en Oost-Gelderland | pre-pandemic | 2.969 |
|  | post-pandemic | 3.88 |
|  | change | 0.911 |
| Noord-Holland-Noord | pre-pandemic | 2.736 |
|  | post-pandemic | 3.509 |
|  | change | 0.773 |
| Rotterdam-Rijnmond | pre-pandemic | 2.904 |
|  | post-pandemic | 4.352 |
|  | change | 1.447 |
| Twente | pre-pandemic | 3.421 |
|  | post-pandemic | 4.007 |
|  | change | 0.585 |
| Utrecht | pre-pandemic | 3.056 |
|  | post-pandemic | 4.148 |
|  | change | 1.092 |
| Zaanstreek-Waterland | pre-pandemic | 3.12 |
|  | post-pandemic | 4.005 |
|  | change | 0.885 |
| Zeeland | pre-pandemic | 3.184 |
|  | post-pandemic | 4.927 |
|  | change | 1.742 |
| Zuid-Holland-Zuid | pre-pandemic | 2.934 |
|  | post-pandemic | 4.069 |
|  | change | 1.135 |

**Table S13***:* Pearson correlations between Kendall’s τ of the DIORs and the change in long-term sick leave rates across safety regions. The table displays the Pearson correlation coefficients and corresponding p-value of the Kendall’s τ of the autocorrelation at lag-1 (ACF1) and standard deviation (SD) estimates during the pandemic on the long-term sick leave data after Gaussian detrending (bandwidths 3-20)*.* Significant correlations are indicated in bold.

| **Detrending** | **ACF1** | | **SD** | |
| --- | --- | --- | --- | --- |
|  | Pearson *r* change sick-leave | p-value | Pearson *r* change sick-leave | p-value |
| *Bandwidths* |  |  |  |  |
| 3 | 0.207 | 0.321 | 0.123 | 0.557 |
| 4 | 0.181 | 0.386 | 0.131 | 0.532 |
| 5 | 0.126 | 0.549 | 0.136 | 0.516 |
| 6 | 0.118 | 0.575 | 0.188 | 0.367 |
| 7 | 0.137 | 0.514 | 0.185 | 0.375 |
| 8 | 0.129 | 0.539 | 0.237 | 0.254 |
| 9 | 0.115 | 0.586 | 0.270 | 0.192 |
| 10 | 0.119 | 0.573 | 0.287 | 0.164 |
| 11 | 0.100 | 0.636 | 0.307 | 0.135 |
| 12 | 0.105 | 0.617 | 0.312 | 0.129 |
| 13 | 0.095 | 0.65 | 0.313 | 0.128 |
| 14 | 0.105 | 0.619 | 0.316 | 0.124 |
| 15 | 0.098 | 0.643 | 0.323 | 0.115 |
| 16 | 0.085 | 0.685 | 0.323 | 0.115 |
| 17 | 0.083 | 0.693 | 0.312 | 0.128 |
| 18 | 0.086 | 0.682 | 0.311 | 0.130 |
| 19 | 0.077 | 0.715 | 0.293 | 0.155 |
| 20 | 0.075 | 0.721 | 0.296 | 0.151 |

* P values < .05 were considered significant

**Table S14***:* Results of the questionnaire for the most relevant indicator of healthcare availability. The selected indicator is indicated in bold.

| **Indicators** | **Count** | **Notes:** |
| --- | --- | --- |
| Pressure on follow-up care (number of patients waiting for geriatric rehabilitation, general practitioner care, paramedical and district nursing) | 28 | Data not publicly available |
| Press the clinic | 23 | Data not publicly available |
| **Absenteeism due to illness in healthcare (short-term (1/to 91 days) and long-term (92 to 730 days))** | 18 | Data publicly available |
| Waiting time per type of care (urgent care, critical plannable care and plannable care) | 18 | Data not publicly available |
| The percentage of hospitals scaling down in critically plannable care (Urgency class 3) | 15 | Data not publicly available |
| Pressure on ICU | 14 |  |
| Availability of ICU beds | 12 |  |
| Registration waiting times (in weeks) for mental health care (independent and institutional) | 11 |  |
| Waiting time per healthcare sector | 10 |  |
| The percentage of hospitals scaling down in urgent care (Urgency class 1 and 2) | 9 |  |
| The percentage of hospitals scaling down in plannable care (Urgency classes 4 and 5) | 9 |  |
| Waiting time per specialism | 9 |  |
| The number of postponed surgeries | 9 |  |
| The percentage of occupied long-term care beds by care healthcare sector | 8 |  |
| The number of postponed surgeries per specialism (increase in the number of regular working weeks and number of surgeries per week) | 6 |  |
| Total number of surgeries (invasive and intensive procedures) performed weekly | 5 |  |
| Waiting time by type of consultation (treatment, diagnostics or outpatient clinic visit) | 4 |  |
| The number of non-urgent surgeries (Urgency Class 4 and 5) performed weekly | 3 |  |
| Total number of patients in care per week in hospitals (minimum outpatient contact) | 3 |  |
| The percentage of closed operating theatres | 2 |  |
| The number of plannable operations (Urgency Class 3) performed weekly | 2 |  |
| The number of urgent surgeries (Urgency Class 1 and 2) performed weekly | 2 |  |
| The national number of referrals to hospitals and independent treatment centres (ZBC) | 2 |  |
| Number of referrals for young people to mental health care per week | 2 |  |
| The number of new diagnoses per specialty | 1 |  |


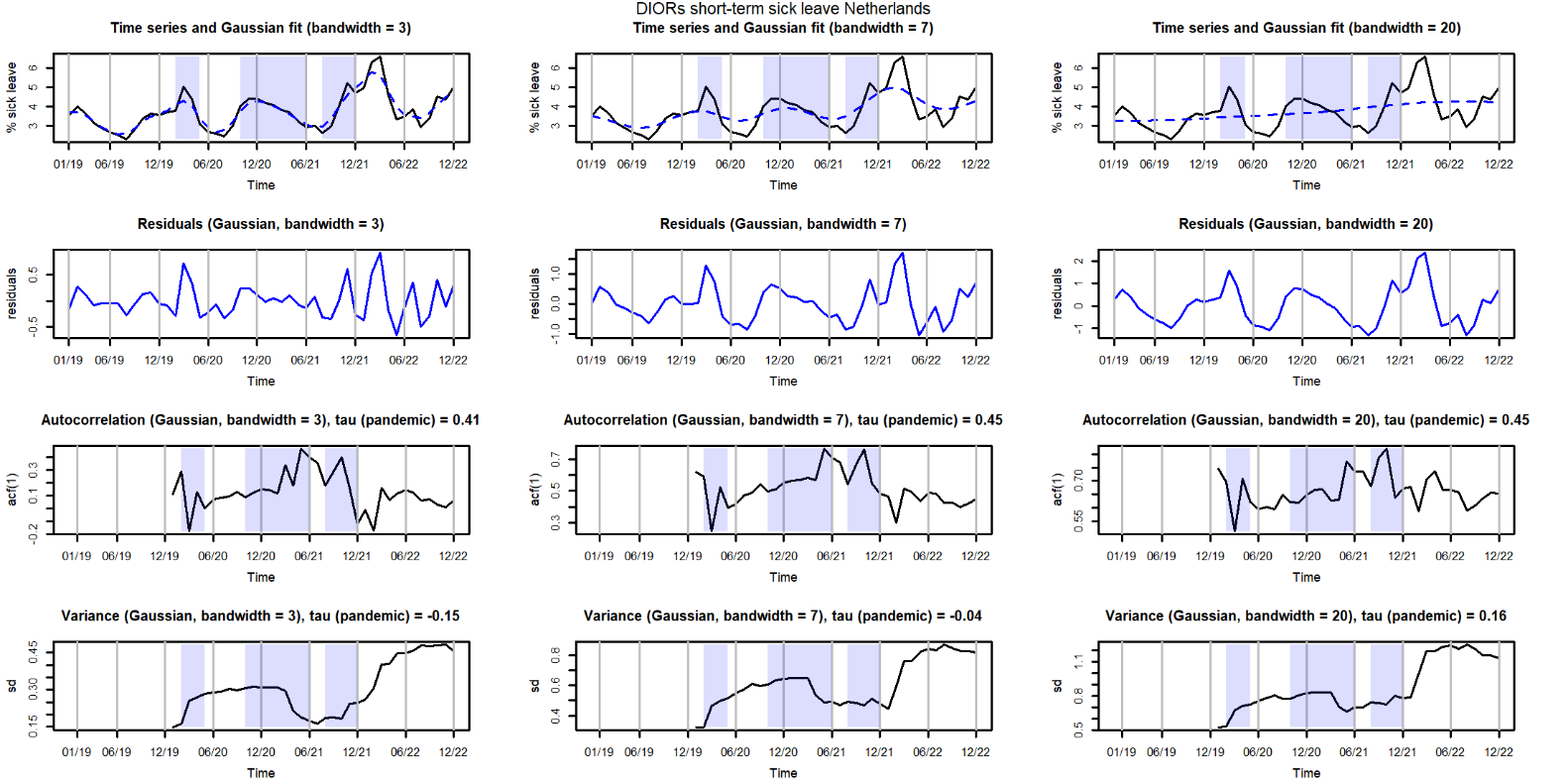
**Figure S1**: Dynamic indicators of resilience for the monthly short-term sick leave rates among healthcare workers in the Netherlands for Gaussian bandwidth = 3 (left column), Gaussian bandwidth = 7 (middle column), Gaussian bandwidth = 20 (right column). The first row shows the original sick leave data (solid line) and the fitted values of the detrending method (blue dotted line). The second row shows data after detrending; i.e., the residuals between the fitted values of the Gaussian detrending curve and original data. The last two rows show the estimated DIORs over time: autocorrelation at lag-1 (ACF1) and standard deviation (SD). The Kendall tau value indicate the trend in DOIRs during the pandemic. The blue coloured bars indicate the periods of the three covid waves.


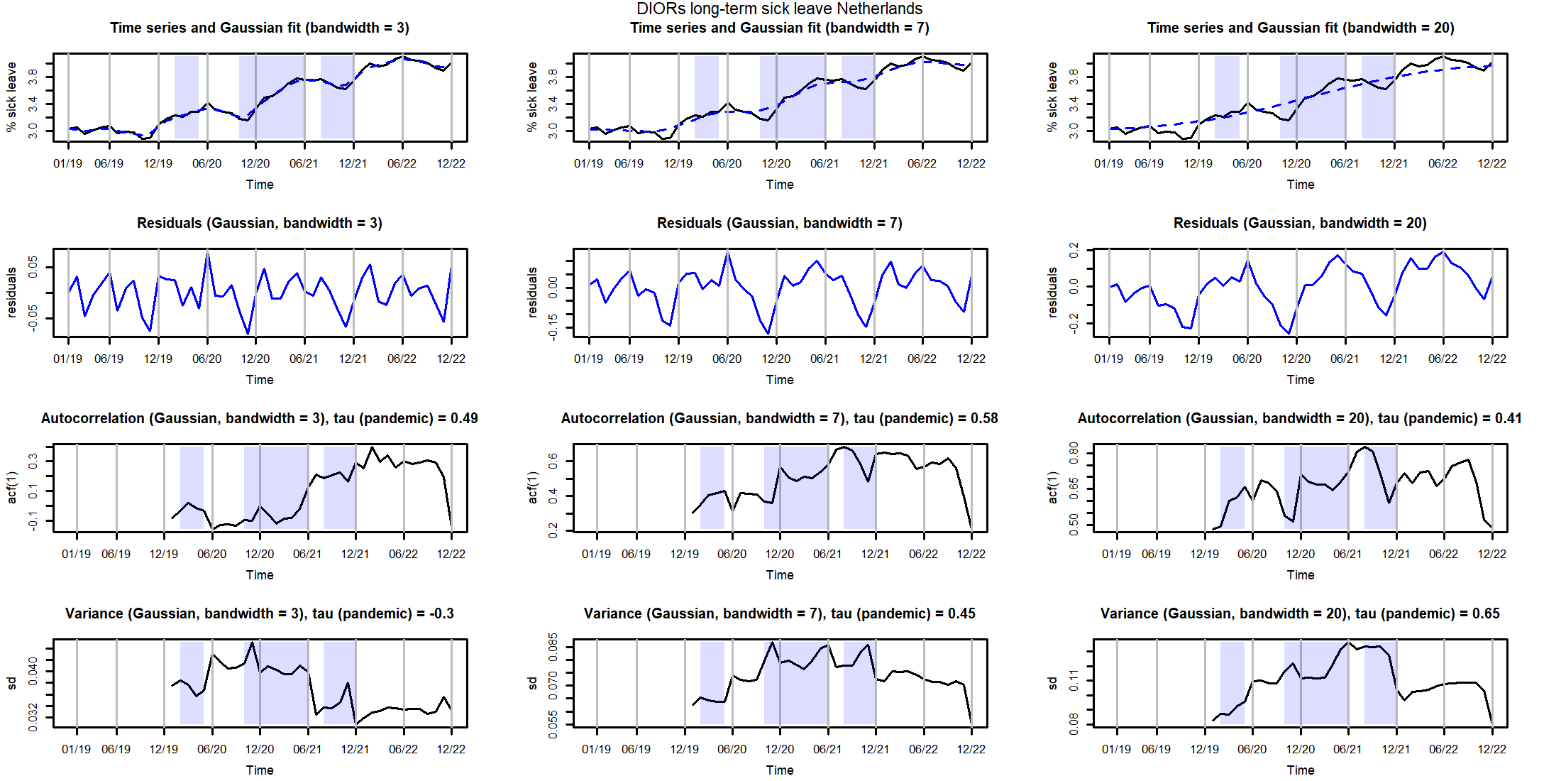
**Figure S2**: Dynamic indicators of resilience for the monthly long-term sick leave rates among healthcare workers in the Netherlands for Gaussian bandwidth = 3 (left column), Gaussian bandwidth = 7 (middle column), Gaussian bandwidth = 20(right column). The first row shows the original sick leave data (solid line) and the fitted values of the detrending method (blue dotted line). The second row shows data after detrending; i.e., the residuals between the fitted values of the Gaussian detrending curve and original data. The last two rows show the estimated DIORs over time: autocorrelation at lag-1 (ACF1) and standard deviation (SD). The Kendall tau value indicate the trend in DOIRs during the pandemic. The blue coloured bars indicate the periods of the three covid waves.


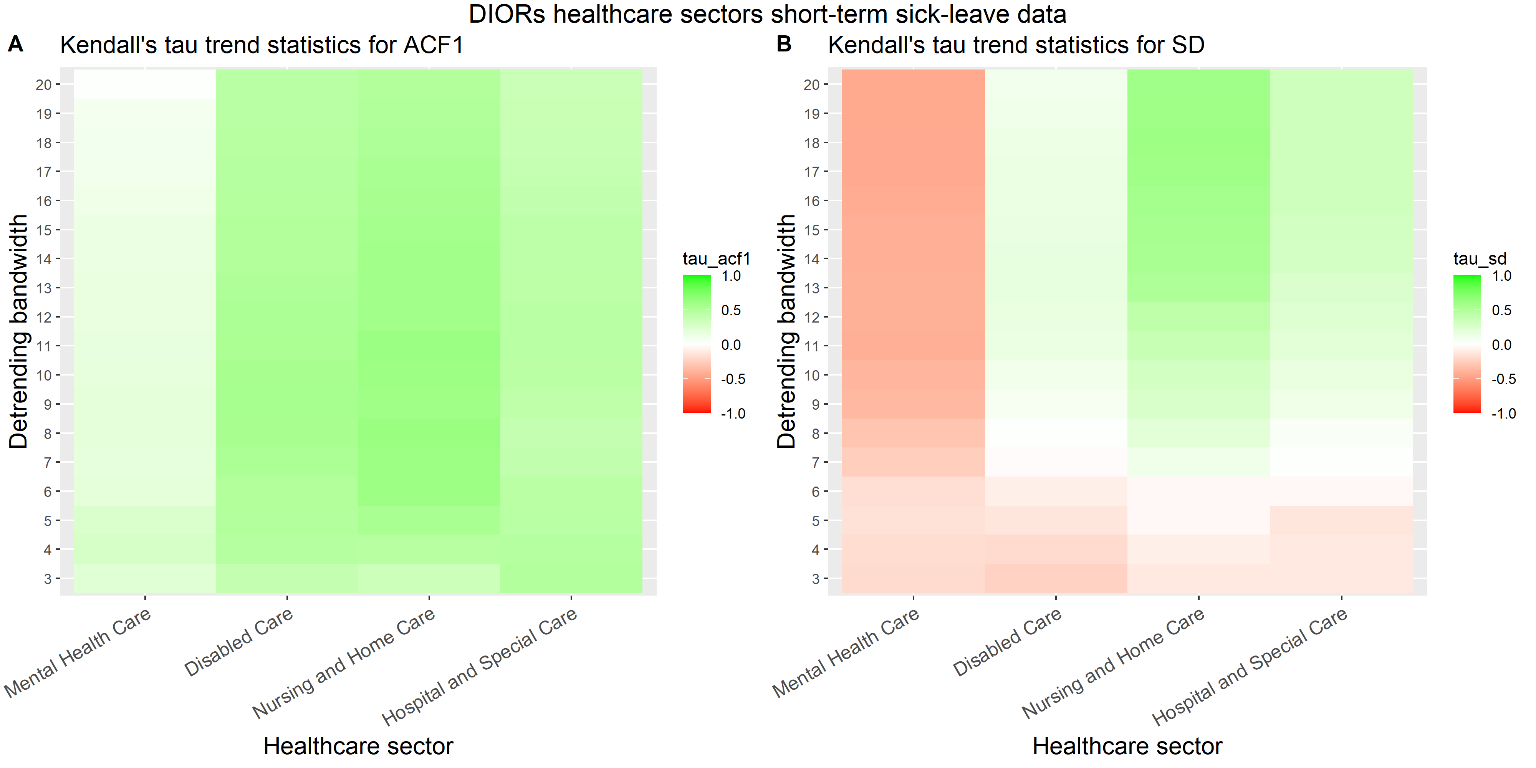
**Figure S3:** Kendall’s τ of DIORs during the pandemic for different bandwidths on the short-term sick-leave rates of each healthcare sectors (March 2020 – December 2021). The left panel gives the Kendall’s τ of the ACF1 estimates, the right panel gives the Kendall’s τ of the SD estimates. Green indicates a positive Kendall’s τ, which indicates an increase in DIORs over time, red indicate a negative Kendall’s τ which indicates a decline in DIORs over time.


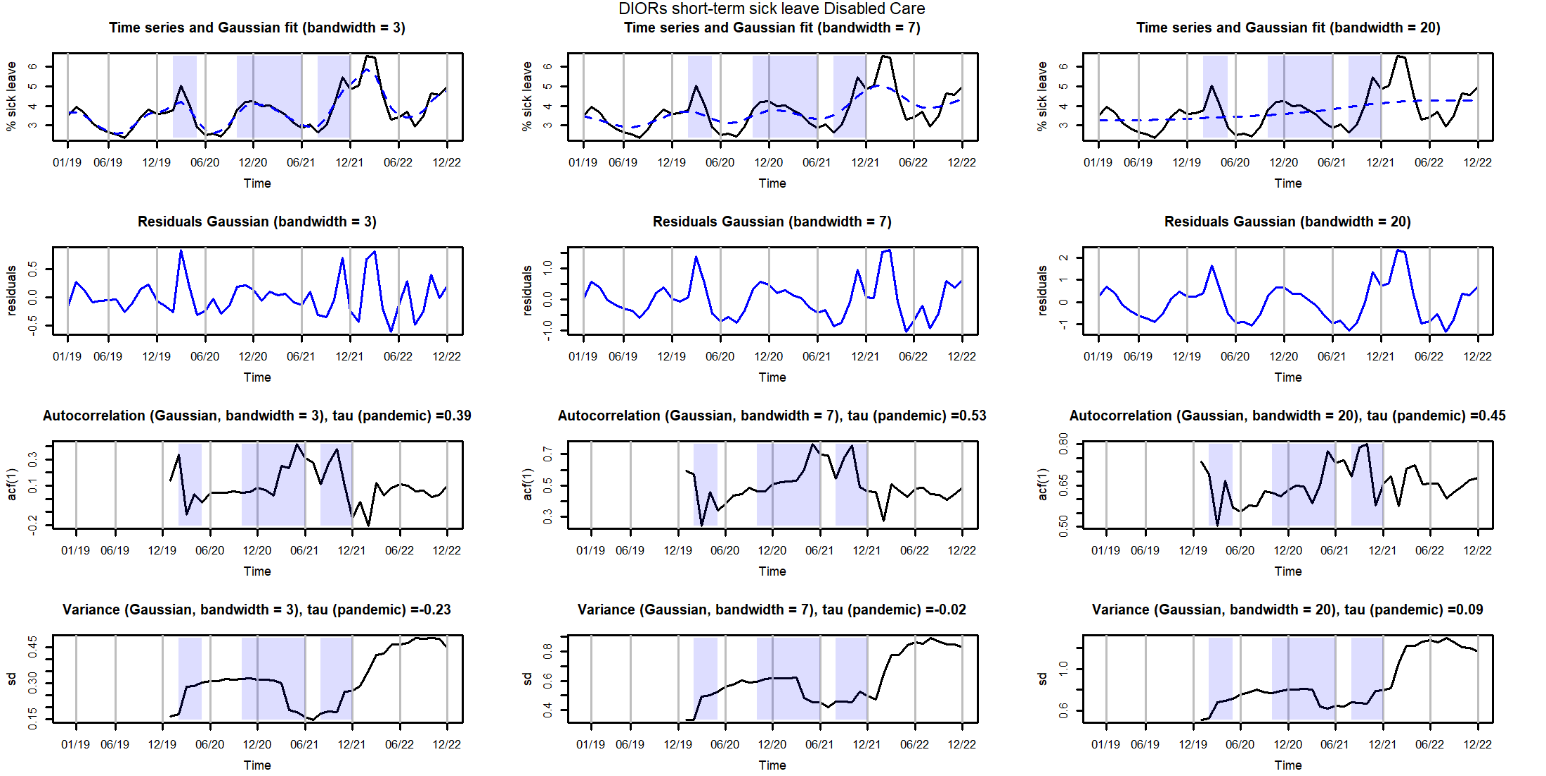


**Figure S4**: Dynamic indicators of resilience for the monthly short-term sick leave rates among healthcare workers in disabled care for Gaussian bandwidth = 3 (left column), Gaussian bandwidth = 7 (middle column), Gaussian bandwidth = 20(right column). The first row shows the original sick leave data (solid line) and the fitted values of the detrending method (blue dotted line). The second row shows data after detrending; i.e., the residuals between the fitted values of the Gaussian detrending curve and original data. The last two rows show the estimated DIORs over time: autocorrelation at lag-1 (ACF1) and standard deviation (SD). The Kendall tau value indicate the trend in DOIRs during the pandemic. The blue coloured bars indicate the periods of the three covid waves.


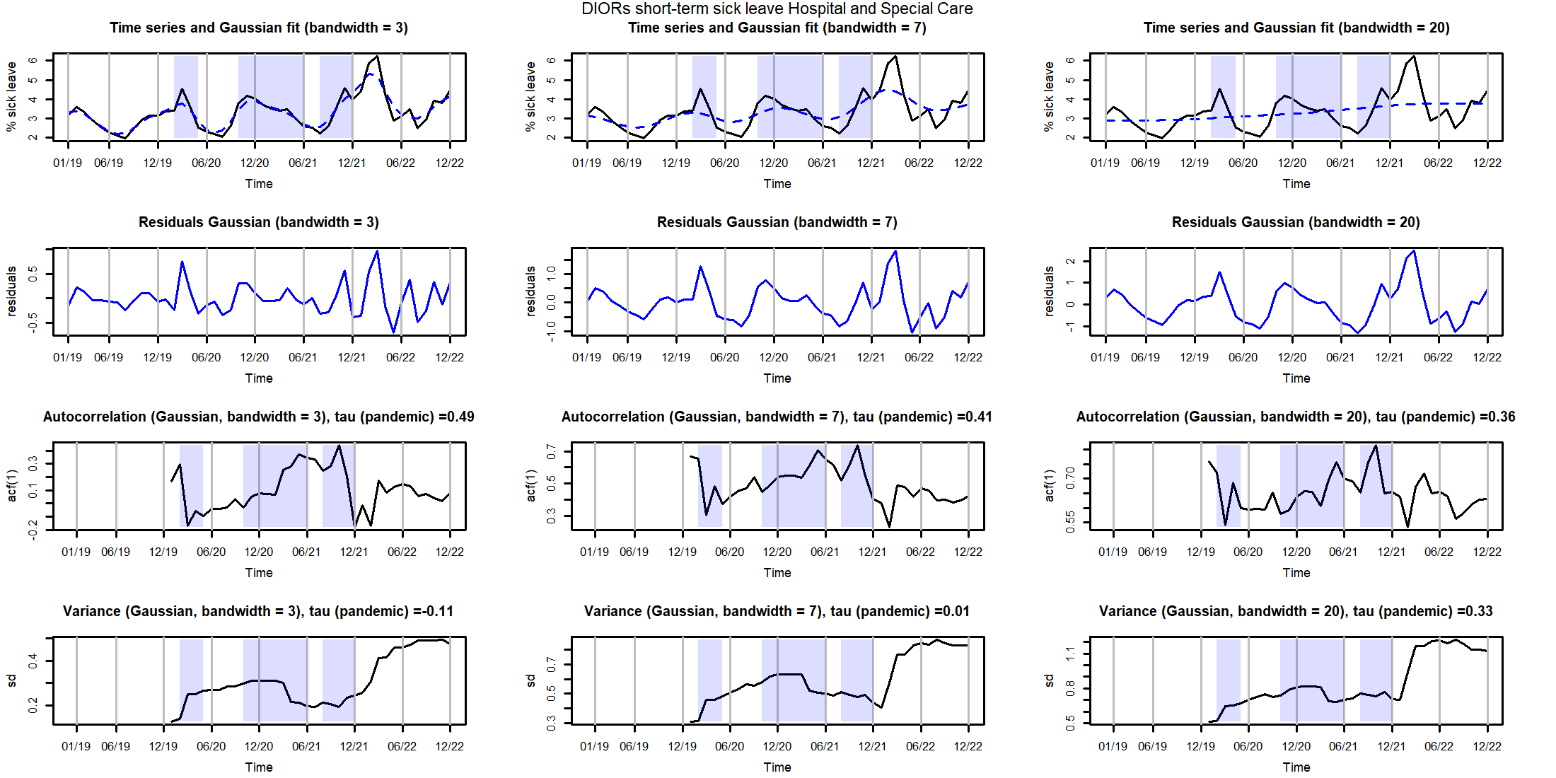


**Figure S5**: Dynamic indicators of resilience for the monthly short-term sick leave rates among healthcare workers in hospital and special care for Gaussian bandwidth = 3 (left column), Gaussian bandwidth = 7 (middle column), Gaussian bandwidth = 20(right column). The first row shows the original sick leave data (solid line) and the fitted values of the detrending method (blue dotted line). The second row shows data after detrending; i.e., the residuals between the fitted values of the Gaussian detrending curve and original data. The last two rows show the estimated DIORs over time: autocorrelation at lag-1 (ACF1) and standard deviation (SD). The Kendall tau value indicate the trend in DOIRs during the pandemic. The blue coloured bars indicate the periods of the three covid waves.


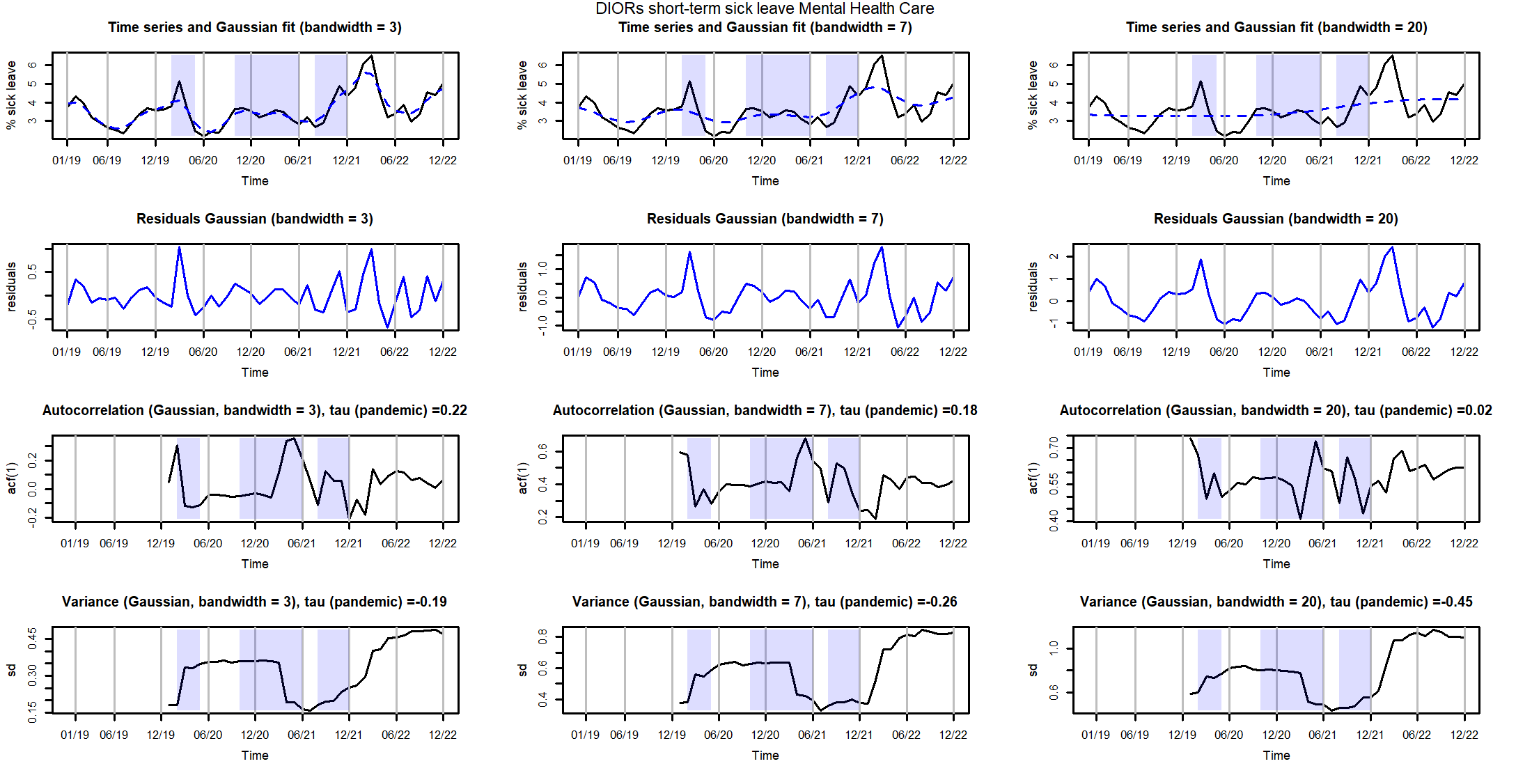


**Figure S6**: Dynamic indicators of resilience for the monthly short-term sick leave rates among healthcare workers in mental healthcare for Gaussian bandwidth = 3 (left column), Gaussian bandwidth = 7 (middle column), Gaussian bandwidth = 20(right column). The first row shows the original sick leave data (solid line) and the fitted values of the detrending method (blue dotted line). The second row shows data after detrending; i.e., the residuals between the fitted values of the Gaussian detrending curve and original data. The last two rows show the estimated DIORs over time: autocorrelation at lag-1 (ACF1) and standard deviation (SD). The Kendall tau value indicate the trend in DOIRs during the pandemic. The blue coloured bars indicate the periods of the three covid waves.


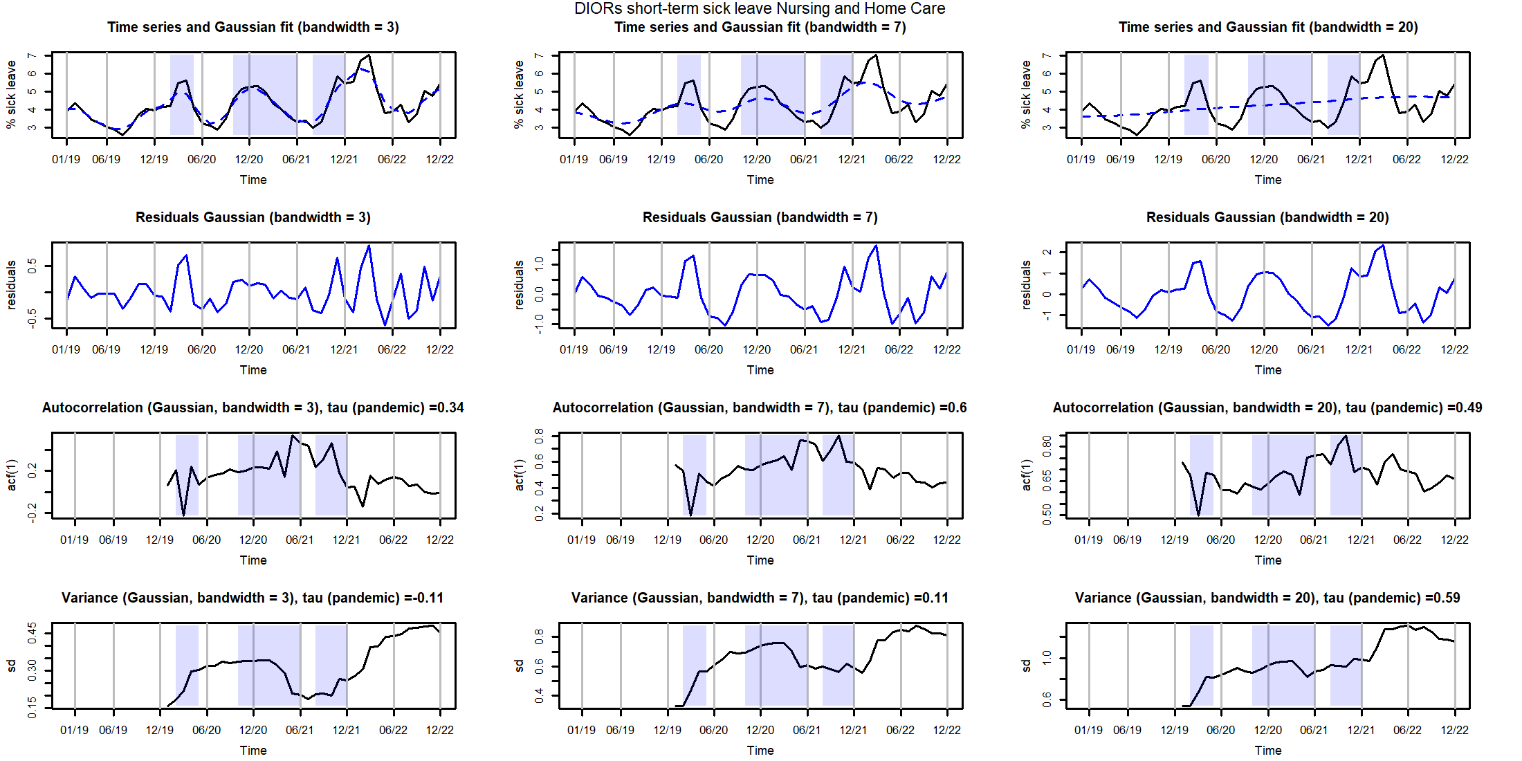


**Figure S7:** Dynamic indicators of resilience for the monthly short-term sick leave rates among healthcare workers in nursing and home care for Gaussian bandwidth = 3 (left column), Gaussian bandwidth = 7 (middle column), Gaussian bandwidth = 20(right column). The first row shows the original sick leave data (solid line) and the fitted values of the detrending method (blue dotted line). The second row shows data after detrending; i.e., the residuals between the fitted values of the Gaussian detrending curve and original data. The last two rows show the estimated DIORs over time: autocorrelation at lag-1 (ACF1) and standard deviation (SD). The Kendall tau value indicate the trend in DOIRs during the pandemic. The blue coloured bars indicate the periods of the three covid waves.


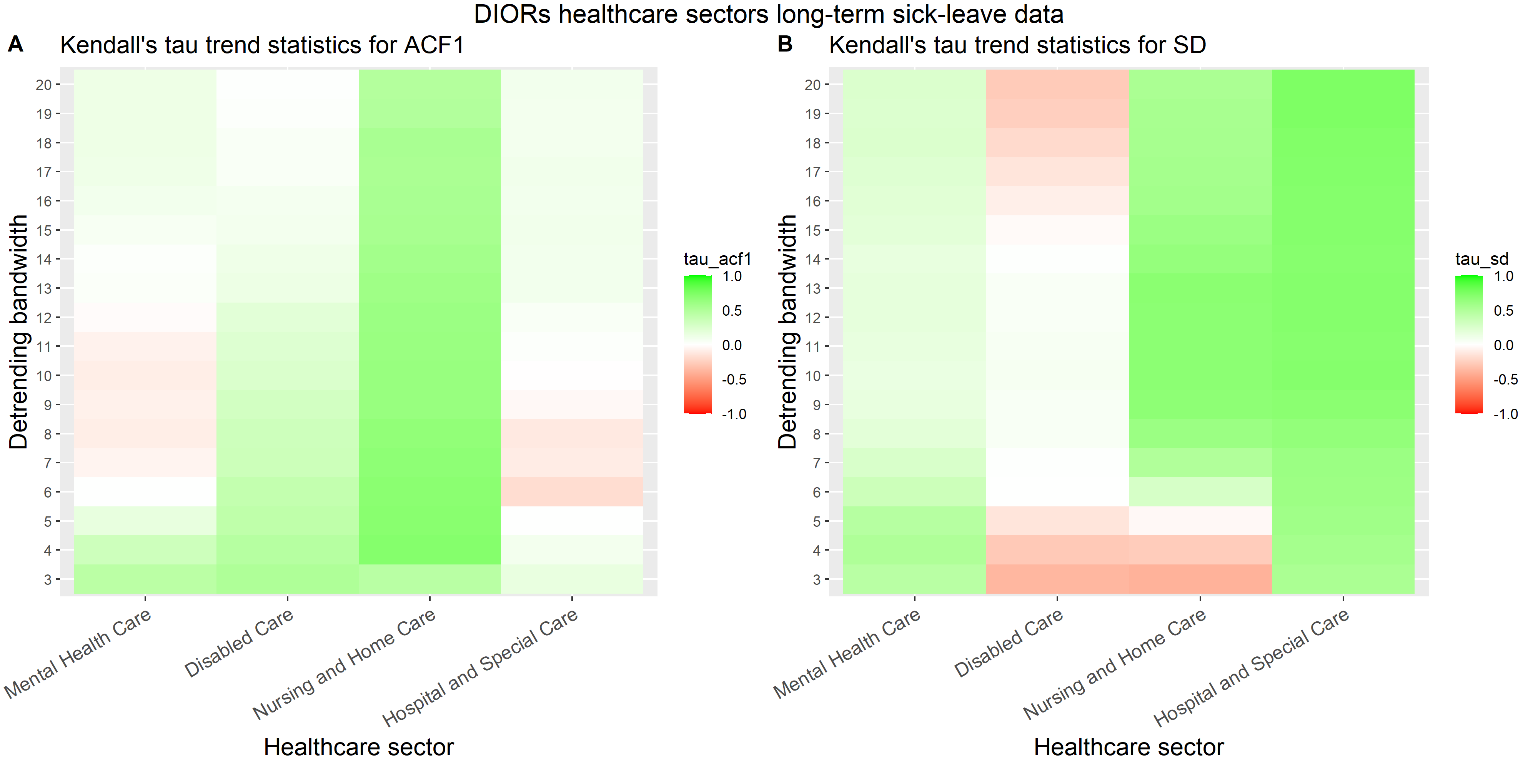
**Figure S8:** Kendall’s τ of DIORs during the pandemic for different bandwidths on the long-term sick-leave rates of each healthcare sectors (March 2020 – December 2021). The left panel gives the Kendall’s τ of the ACF1 estimates, the right panel gives the Kendall’s τ of the SD estimates. Green indicates a positive Kendall’s τ, which indicates an increase in DIORs over time, red indicate a negative Kendall’s τ which indicates a decline in DIORs over time.


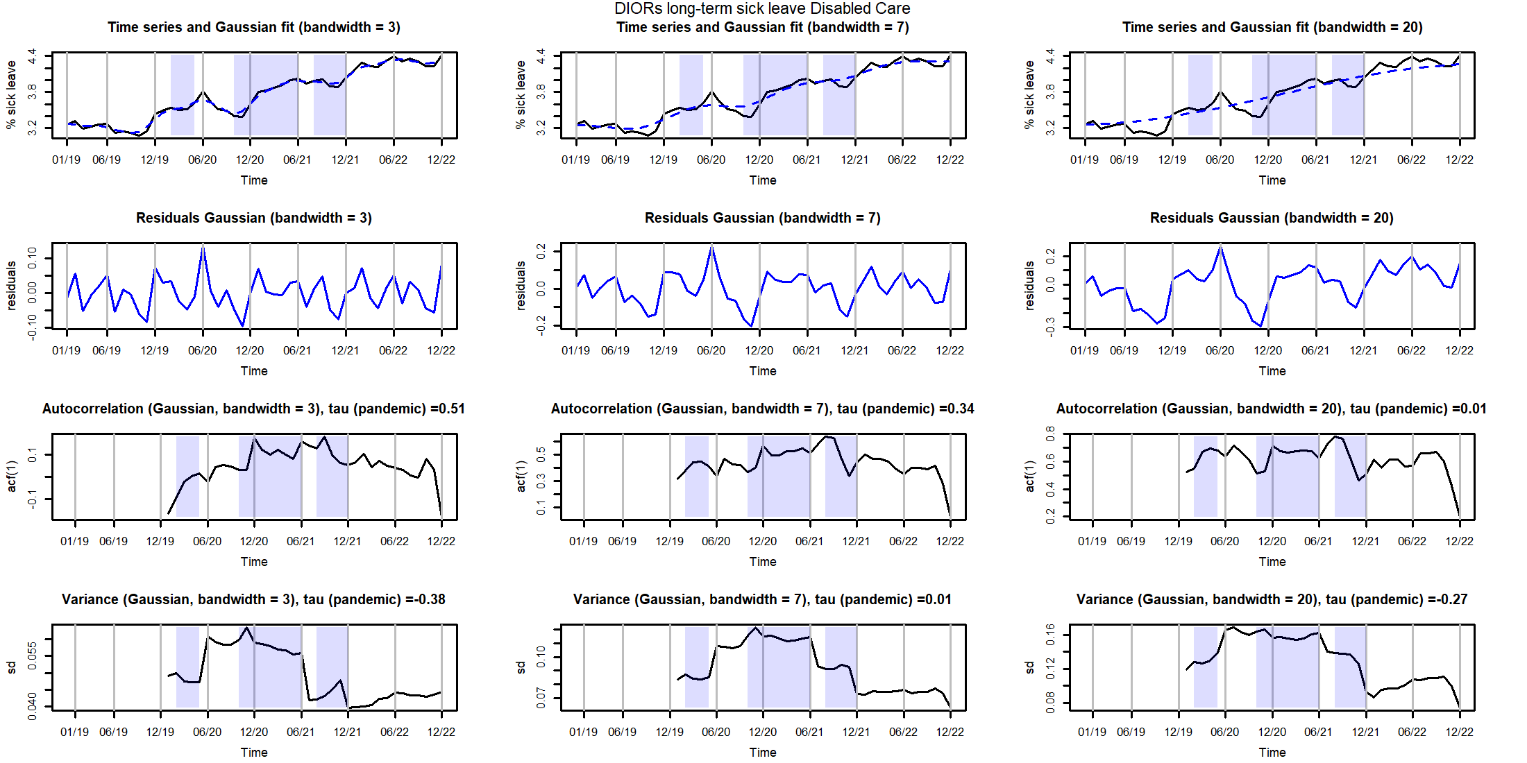
**Figure S9:** Dynamic indicators of resilience for the monthly long -term sick leave rates among healthcare workers in disabled care for Gaussian bandwidth = 3 (left column), Gaussian bandwidth = 7 (middle column), Gaussian bandwidth = 20(right column). The first row shows the original sick leave data (solid line) and the fitted values of the detrending method (blue dotted line). The second row shows data after detrending; i.e., the residuals between the fitted values of the Gaussian detrending curve and original data. The last two rows show the estimated DIORs over time: autocorrelation at lag-1 (ACF1) and standard deviation (SD). The Kendall tau value indicate the trend in DOIRs during the pandemic. The blue coloured bars indicate the periods of the three covid waves.


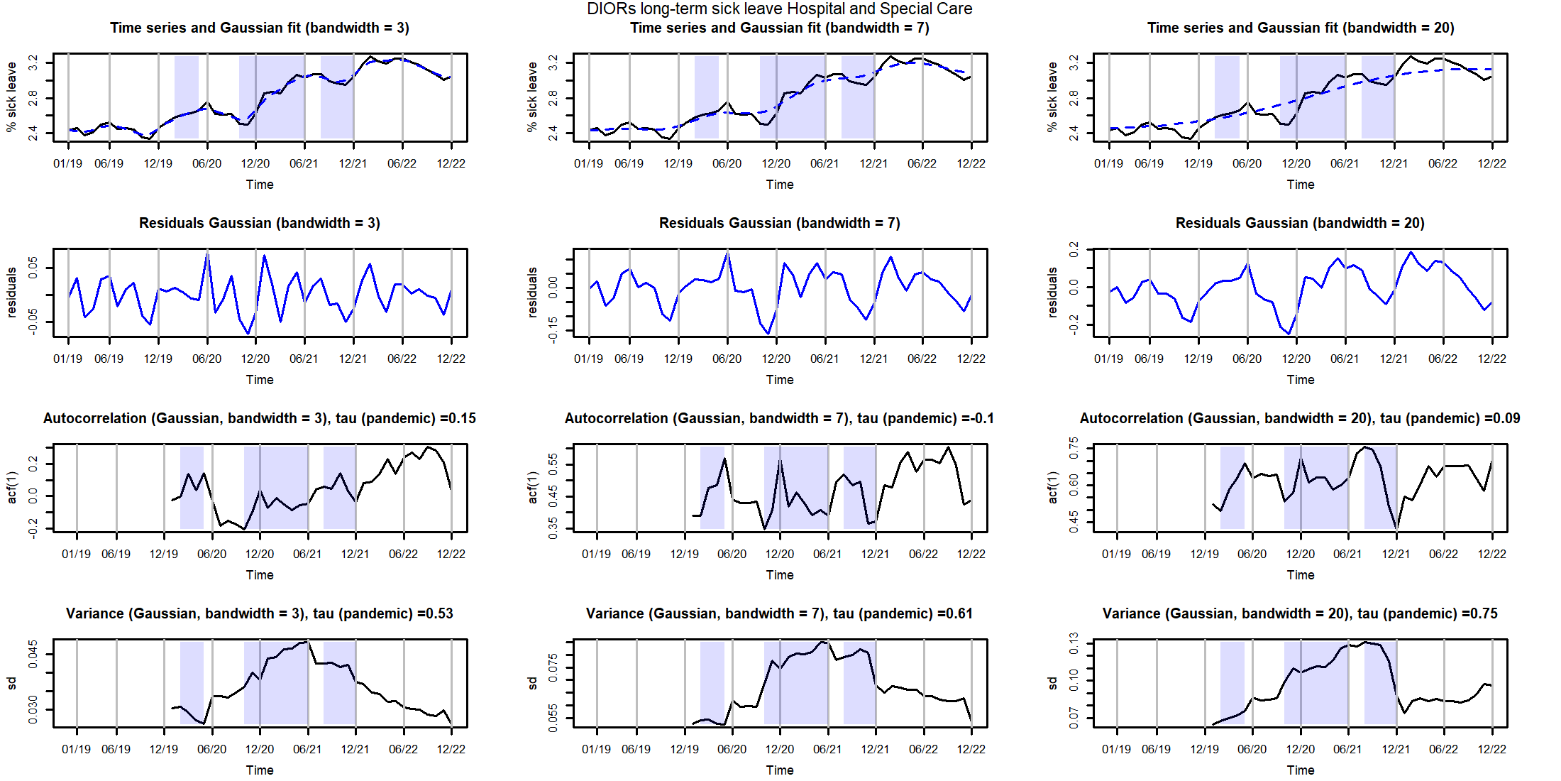
**Figure S10:** Dynamic indicators of resilience for the monthly long -term sick leave rates among healthcare workers in hospital and special care for Gaussian bandwidth = 3 (left column), Gaussian bandwidth = 7 (middle column), Gaussian bandwidth = 20(right column). The first row shows the original sick leave data (solid line) and the fitted values of the detrending method (blue dotted line). The second row shows data after detrending; i.e., the residuals between the fitted values of the Gaussian detrending curve and original data. The last two rows show the estimated DIORs over time: autocorrelation at lag-1 (ACF1) and standard deviation (SD). The Kendall tau value indicate the trend in DOIRs during the pandemic. The blue coloured bars indicate the periods of the three covid waves.


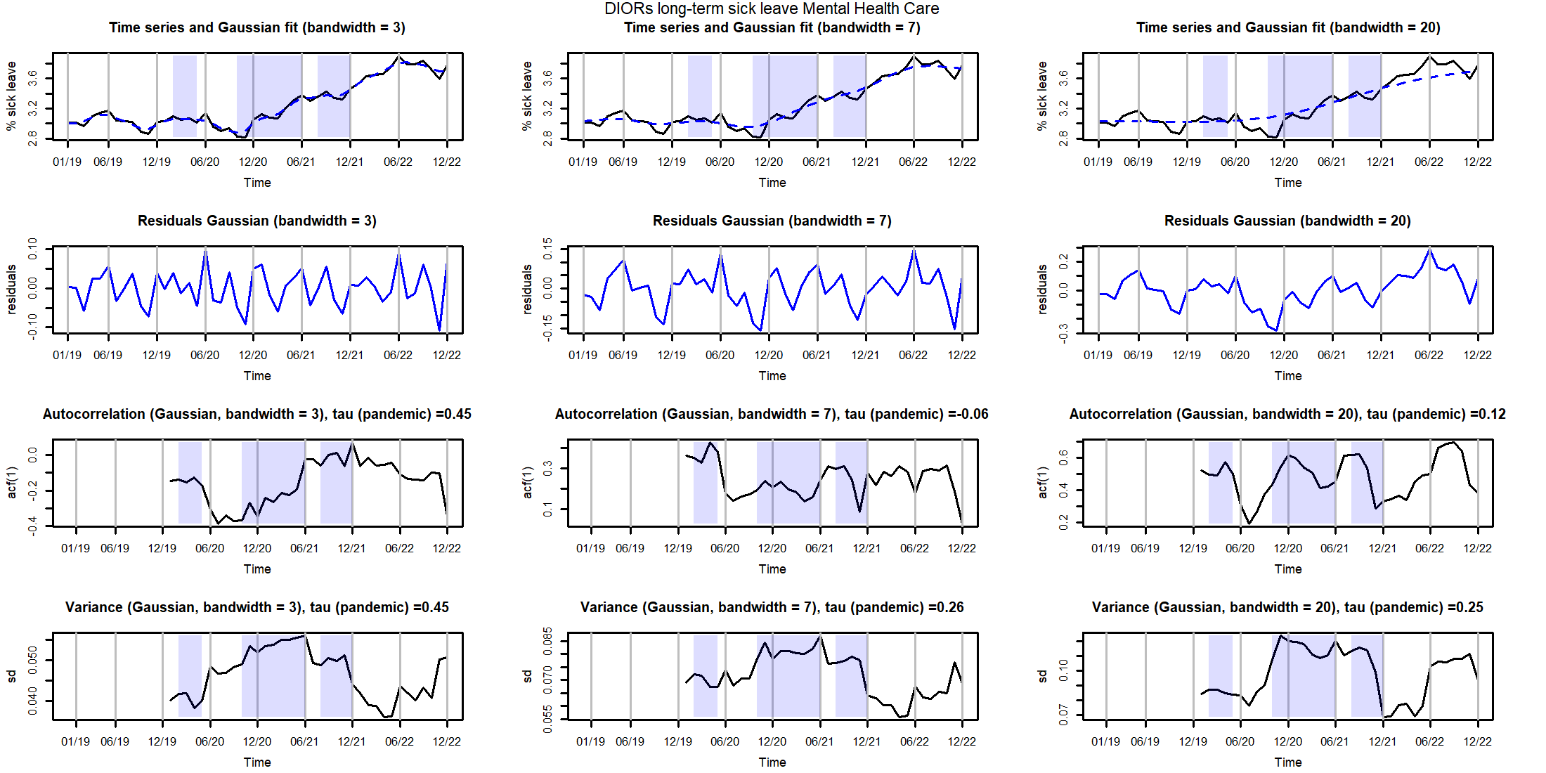


**Figure S11:** Dynamic indicators of resilience for the monthly long -term sick leave rates among healthcare workers in mental healthcare for Gaussian bandwidth = 3 (left column), Gaussian bandwidth = 7 (middle column), Gaussian bandwidth = 20(right column). The first row shows the original sick leave data (solid line) and the fitted values of the detrending method (blue dotted line). The second row shows data after detrending; i.e., the residuals between the fitted values of the Gaussian detrending curve and original data. The last two rows show the estimated DIORs over time: autocorrelation at lag-1 (ACF1) and standard deviation (SD). The Kendall tau value indicate the trend in DOIRs during the pandemic. The blue coloured bars indicate the periods of the three covid waves.


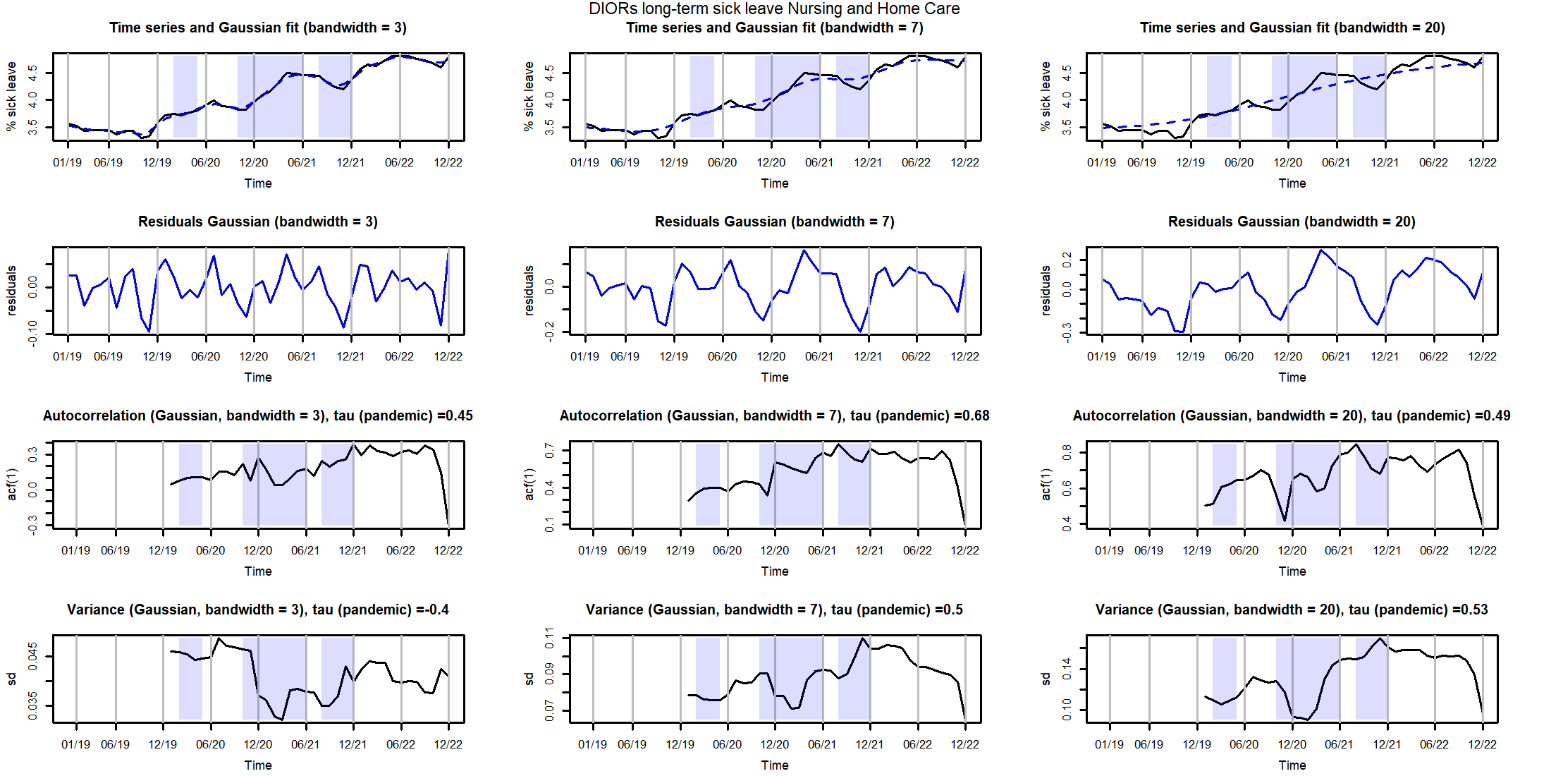


**Figure S12**: Dynamic indicators of resilience for the monthly long -term sick leave rates among healthcare workers in nursing and home care for Gaussian bandwidth = 3 (left column), Gaussian bandwidth = 7 (middle column), Gaussian bandwidth = 20(right column). The first row shows the original sick leave data (solid line) and the fitted values of the detrending method (blue dotted line). The second row shows data after detrending; i.e., the residuals between the fitted values of the Gaussian detrending curve and original data. The last two rows show the estimated DIORs over time: autocorrelation at lag-1 (ACF1) and standard deviation (SD). The Kendall tau value indicate the trend in DOIRs during the pandemic. The blue coloured bars indicate the periods of the three covid waves.


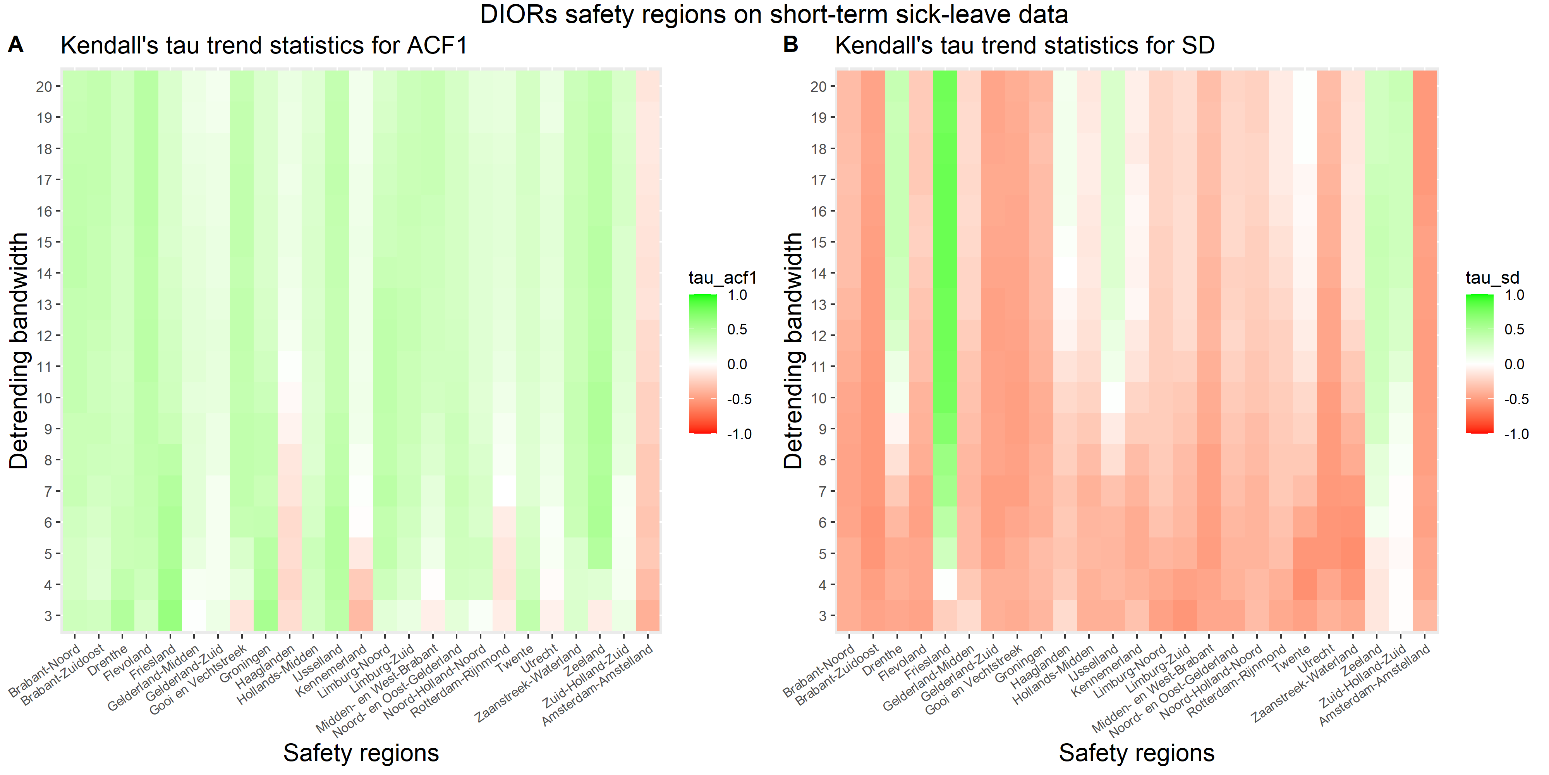
**Figure S13:** Kendall’s τ of DIORs for different bandwidths on the short-term sick-leave rates of each safety region sectors during the pandemic (March 2020 – December 2021). The left panel gives the Kendall’s τ of the ACF1 estimates, the right panel gives the Kendall’s τ of the SD estimates. Green indicates a positive Kendall’s τ, which indicates an increase in DIORs over time, red indicate a negative Kendall’s τ, which indicates a decline in DIORs over time.


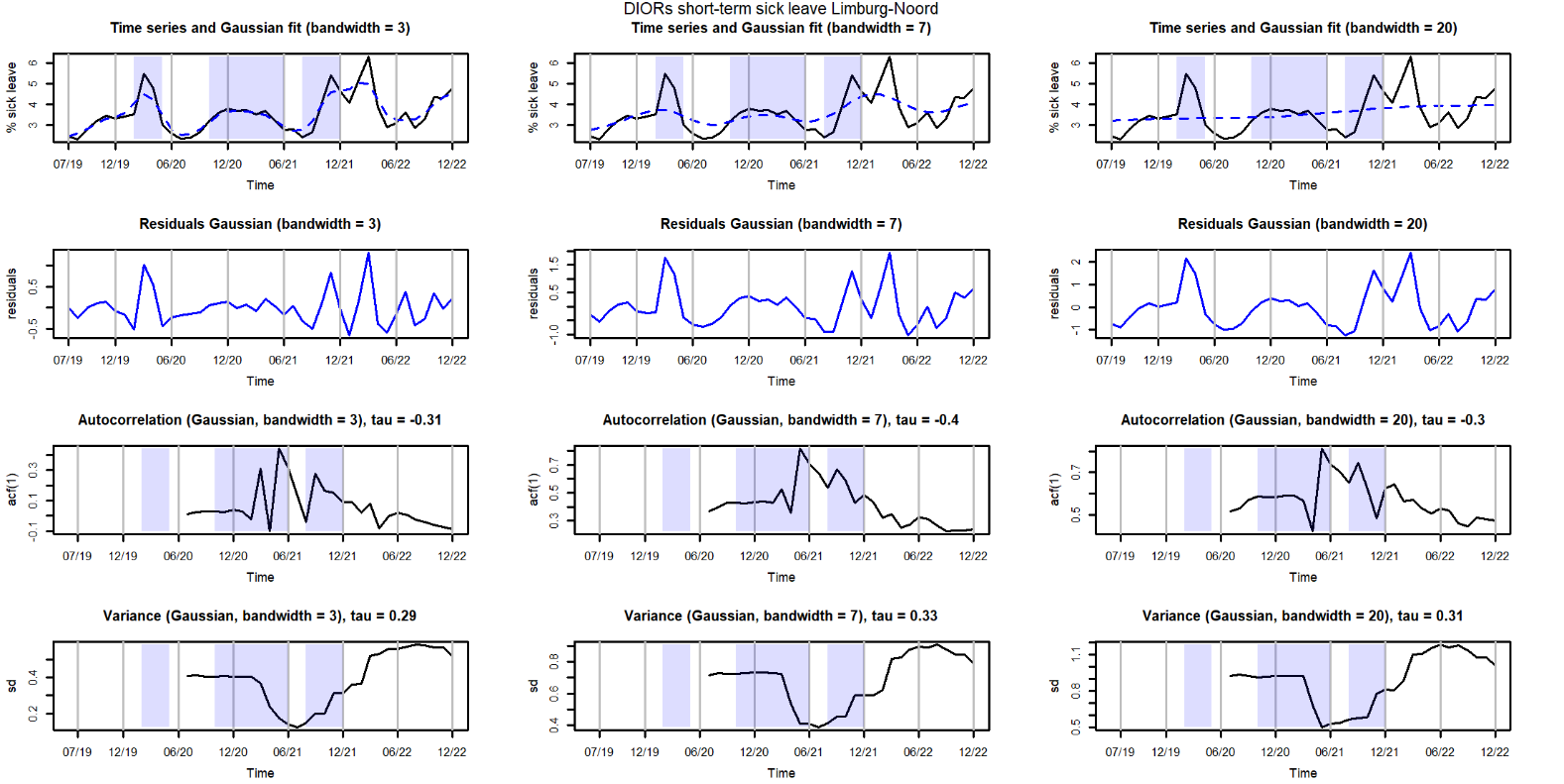
**Figure S14**: Dynamic indicators of resilience for the monthly short -term sick leave rates among healthcare workers in Limburg-Noord for Gaussian bandwidth = 3 (left column), Gaussian bandwidth = 7 (middle column), Gaussian bandwidth = 20(right column). The first row shows the original sick leave data (solid line) and the fitted values of the detrending method (blue dotted line). The second row shows data after detrending; i.e., the residuals between the fitted values of the Gaussian detrending curve and original data. The last two rows show the estimated DIORs over time: autocorrelation at lag-1 (ACF1) and standard deviation (SD). The Kendall tau value indicate the trend in DOIRs during the pandemic. The blue coloured bars indicate the periods of the three covid waves.


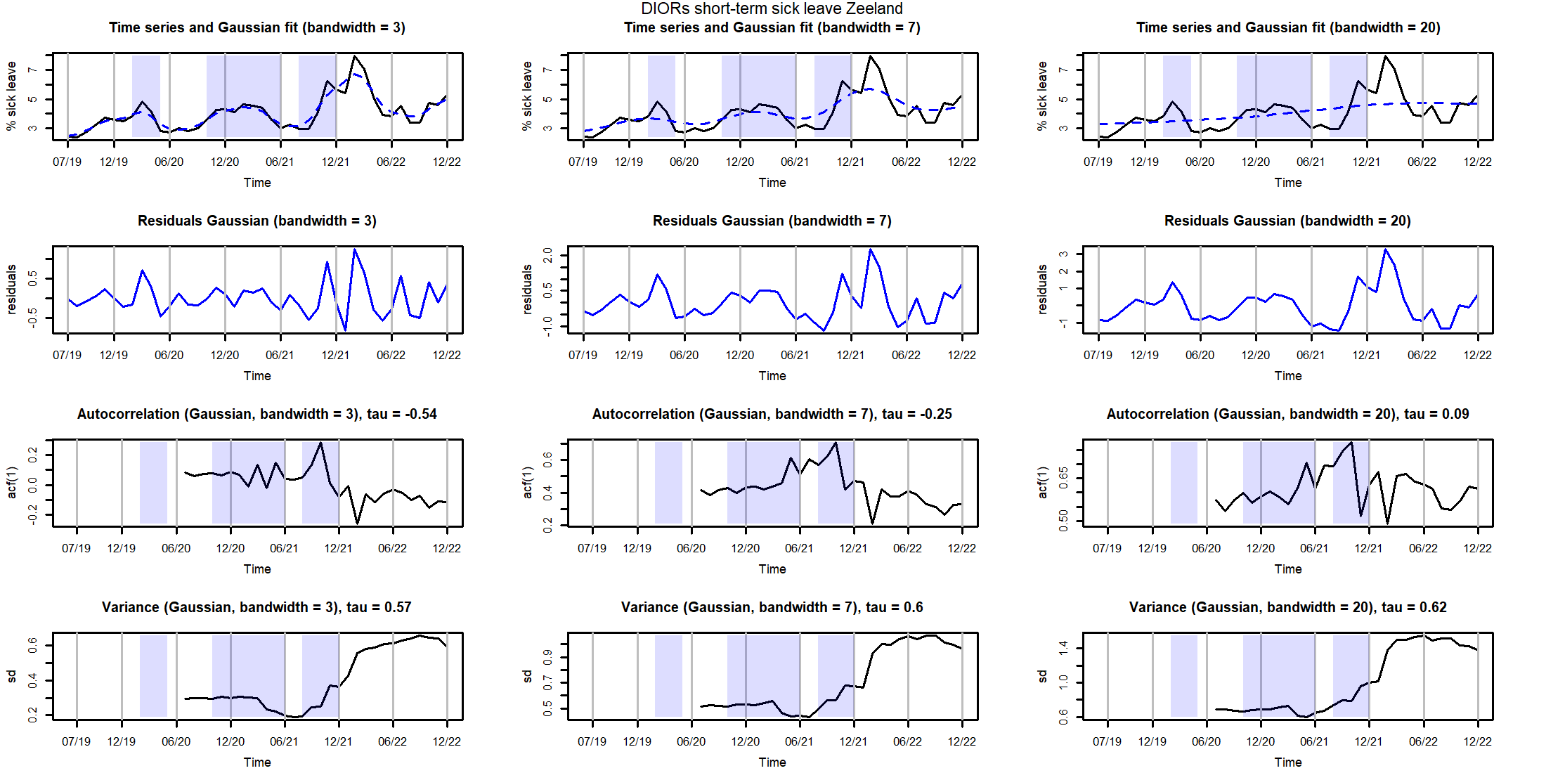
**Figure S15**: Dynamic indicators of resilience for the monthly short -term sick leave rates among healthcare workers in Zeeland for Gaussian bandwidth = 3 (left column), Gaussian bandwidth = 7 (middle column), Gaussian bandwidth = 20(right column). The first row shows the original sick leave data (solid line) and the fitted values of the detrending method (blue dotted line). The second row shows data after detrending; i.e., the residuals between the fitted values of the Gaussian detrending curve and original data. The last two rows show the estimated DIORs over time: autocorrelation at lag-1 (ACF1) and standard deviation (SD). The Kendall tau value indicate the trend in DOIRs during the pandemic. The blue coloured bars indicate the periods of the three covid waves.


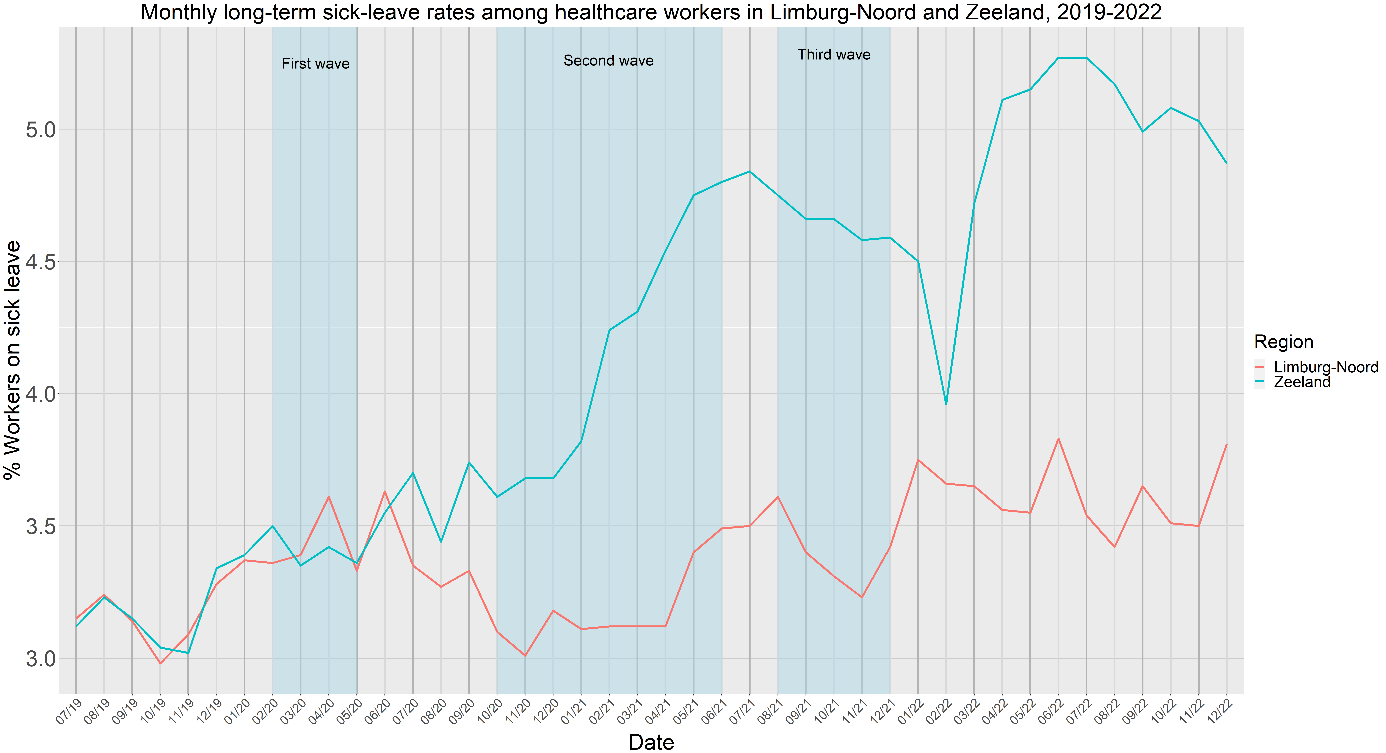
 **Figure S16:** Monthly long-term sick-leave rates (% sick-leave absenteeism among healthcare that takes more than 90 days) among healthcare workers in safety regions Limburg-Noord and Zeeland from July 2019 to December 2022. The three blue vertical bars indicate the time periods of the first, second and third COVID-19 waves.


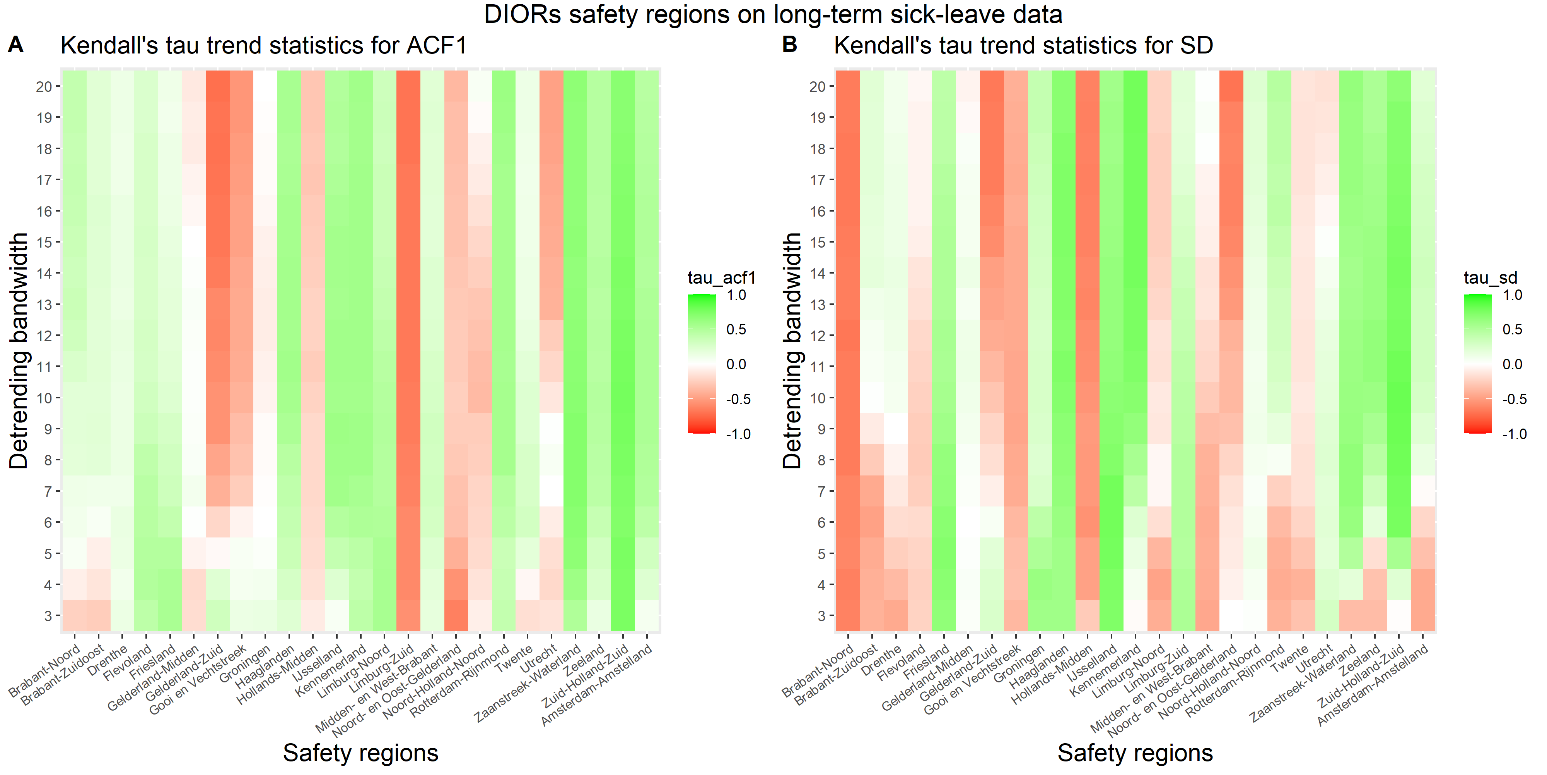
**Figure S17:** Kendall’s τ of DIORs for different bandwidths on the short-term sick-leave rates of each safety region sectors during the pandemic (March 2020 – December 2021). The left panel gives the Kendall’s τ of the ACF1 estimates, the right panel gives the Kendall’s τ of the SD estimates. Green indicates a positive Kendall’s τ, which indicates an increase in DIORs over time, red indicate a negative Kendall’s τ, which indicates a decline in DIORs over time.


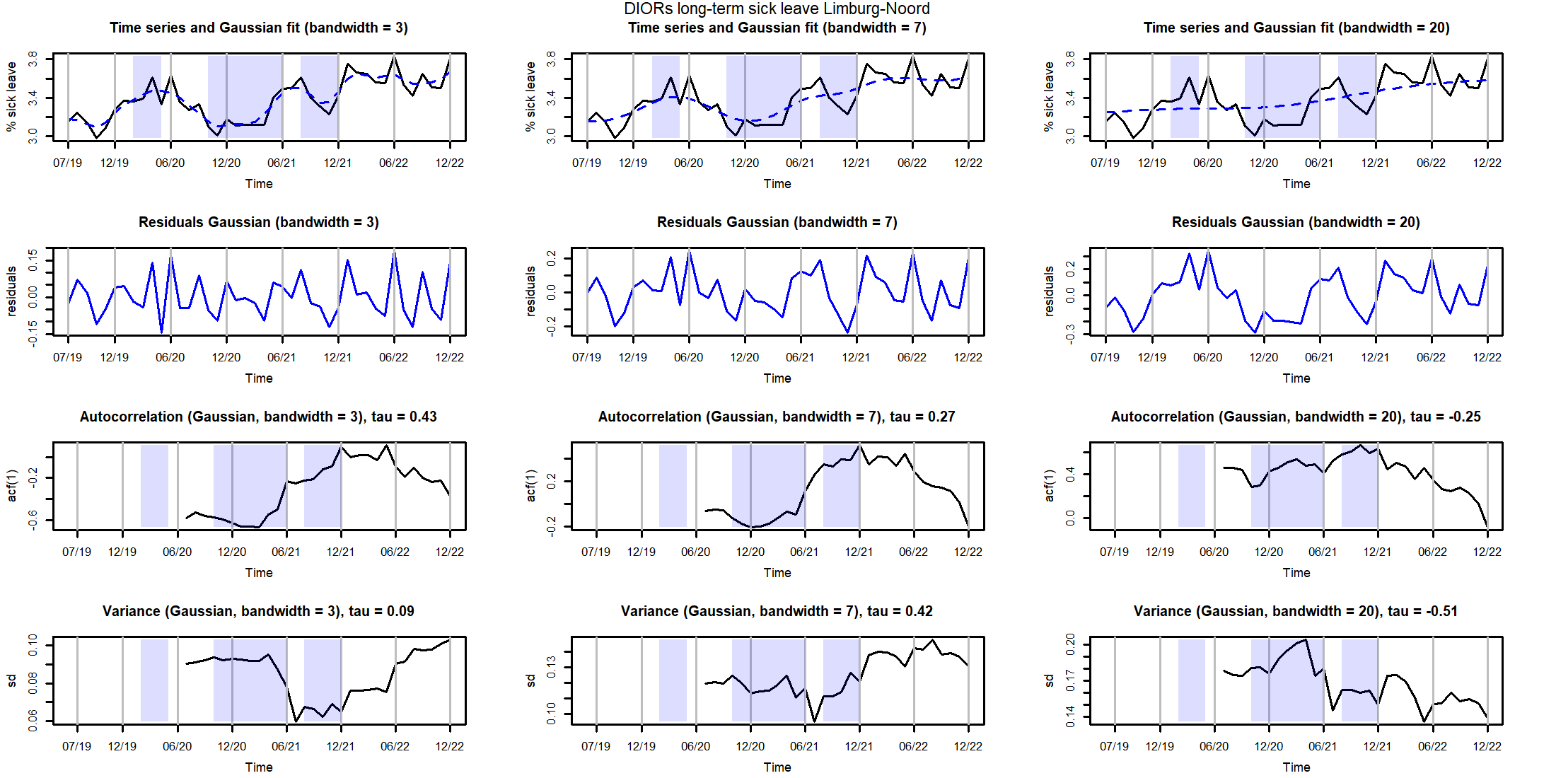
**Figure S18**: Dynamic indicators of resilience for the monthly long -term sick leave rates among healthcare workers in Limburg-Noord for Gaussian bandwidth = 3 (left column), Gaussian bandwidth = 7 (middle column), Gaussian bandwidth = 20(right column). The first row shows the original sick leave data (solid line) and the fitted values of the detrending method (blue dotted line). The second row shows data after detrending; i.e., the residuals between the fitted values of the Gaussian detrending curve and original data. The last two rows show the estimated DIORs over time: autocorrelation at lag-1 (ACF1) and standard deviation (SD). The Kendall tau value indicate the trend in DOIRs during the pandemic. The blue coloured bars indicate the periods of the three covid waves.


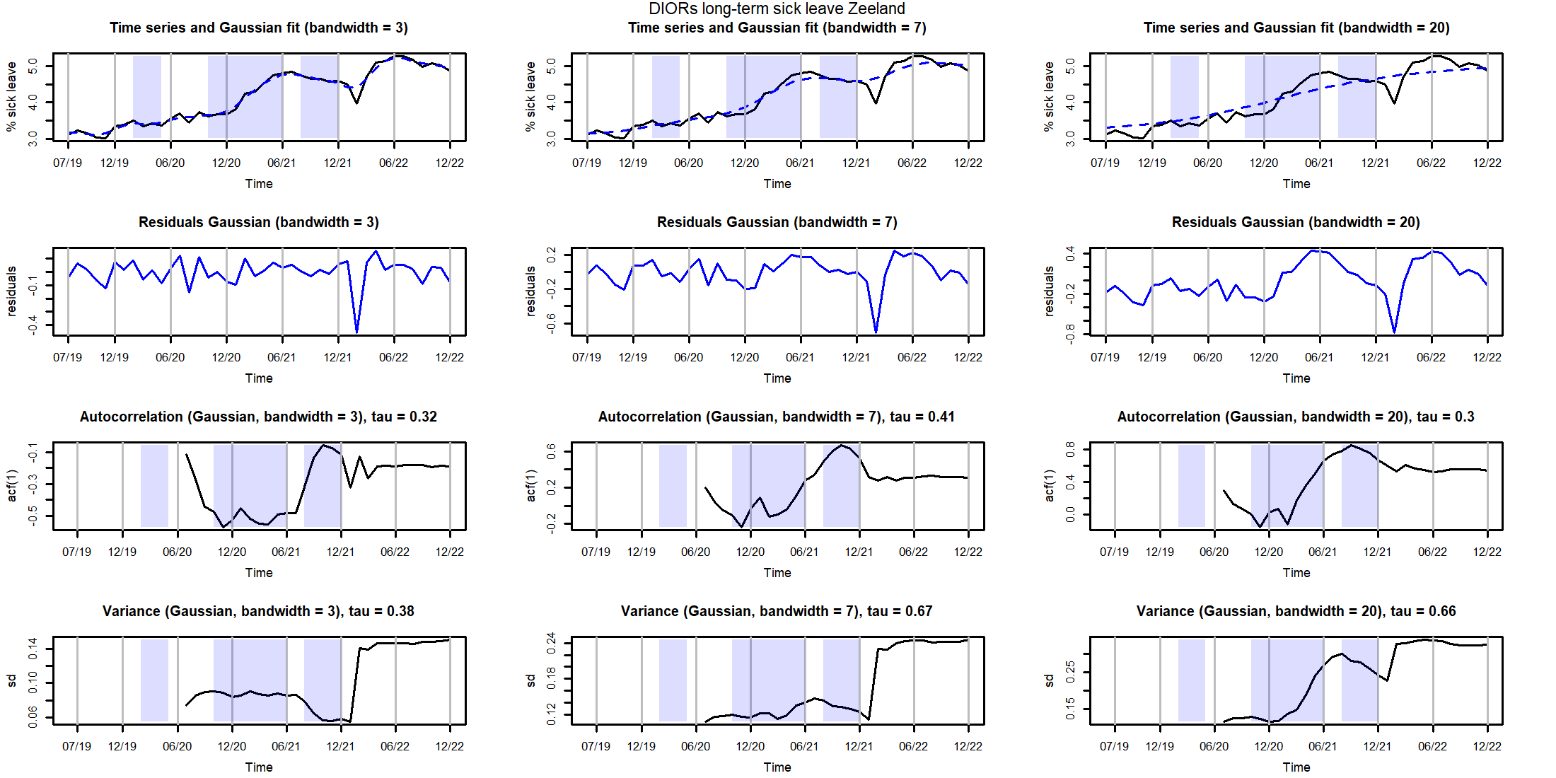
**Figure S19**: Dynamic indicators of resilience for the monthly long -term sick leave rates among healthcare workers in Zeeland for Gaussian bandwidth = 3 (left column), Gaussian bandwidth = 7 (middle column), Gaussian bandwidth = 20(right column). The first row shows the original sick leave data (solid line) and the fitted values of the detrending method (blue dotted line). The second row shows data after detrending; i.e., the residuals between the fitted values of the Gaussian detrending curve and original data. The last two rows show the estimated DIORs over time: autocorrelation at lag-1 (ACF1) and standard deviation (SD). The Kendall tau value indicate the trend in DOIRs during the pandemic. The blue coloured bars indicate the periods of the three covid waves.

**SUPPLEMENTARY MATERIALS: DETRAILED DESCRIPTION OF THE DETRENDING METHOD AND ROBUSTNESS AND SENSITIVITY ANALYSIS**

**Detrending**

Data was detrended using a Gaussian smoothing function that approximates the (non-linear) shape of the trend. The residuals between the fitted values and the observed time-series were used to estimate the DIORs. The shape of the Gaussian smoothing function is determined by averaging each datapoint in the time series with its surrounding datapoints. The width of the Gaussian smoothing function, which determines the degree to which the surrounding datapoints are weighted in the averaging process, is determined by the bandwidth parameter such that quartiles (viewed as probability densities) are at ± 0.25 bandwidth. This means that, the narrower the bandwidth, the narrower the Gaussian function and the more the smoothing function will resemble the original data, as local observations in the time-series are weighted more strongly in the smoothing function. When choosing a bandwidth it is important not to overfit the data (remove all patterns we are interested in) while simultaneously preserve the statistical properties of the patterns (Dessavre et al., 2019). The width of the Gaussian function is scaled such that quartiles (viewed as probability densities) are at ± 0.25 bandwidth, in our study this means that a bandwidth is equal.

**Robustness and sensitivity analysis**

Robustness check predetermined rolling window size

To examine the robustness of the results with the predetermined rolling window size of 27% (i.e., first 13 months before the pandemic), we compared the Kendall’s $\tau$ over time for the ACF1 and SD estimates with arbitrary window sizes [15%, 16%, …, 50% of the data] with bandwidths [3, 6, 7, …, 75] for Gaussian detrending. The robustness check was performed on the nationwide time-series of long- and short-term sick-leave.

Sensitivity analysis detrending method

When detrending the data, it is important not to overfit the data (remove all patterns we are interested in) while simultaneously preserve the statistical properties of the patterns (Dessavre et al., 2019). Sensitivity analysis was therefore conducted to improve the robustness of our results across bandwidths of the Gaussian detrending method. We compared the ACF1 and SD estimates after Gaussian detrending with bandwidths [5, 6, 7, …, 20]. Sensitivity analyses were performed on all analyses (e.g., Kendall’s $\tau$ and Pearson’s *r*) across all datasets (i.e., national, sectors and regional) for both long- and short-term sick-leave.

**SUPPLEMENTARY MATERIALS: DETAILED RESULTS FROM THE ROBUSTNESS AND SENSITIVITY ANALYSIS**

**Short-term sick leave data**

Sensitivity analysis of the window sizes revealed non-robust estimates for ACF1 and robust estimates for the SD (figures S20 and S21). For the ACF1 we found negative Kendall’s τ for smaller window sizes (<11) and positive Kendall’s τ for larger window sizes. For the SD estimates we found consistent positive Kendall’s τ across all window sizes.

**Long-term sick leave data**

Sensitivity analysis of the window sizes revealed robust estimates for both ACF1 and SD (figures S22 and S23). For the ACF1 estimates we found consistent positive Kendall’s τ across all window sizes and bandwidths. For the SD estimates we found consistently low Kendall’s τ, with small negative effects for lower window sizes and small positive effects for larger window sizes.


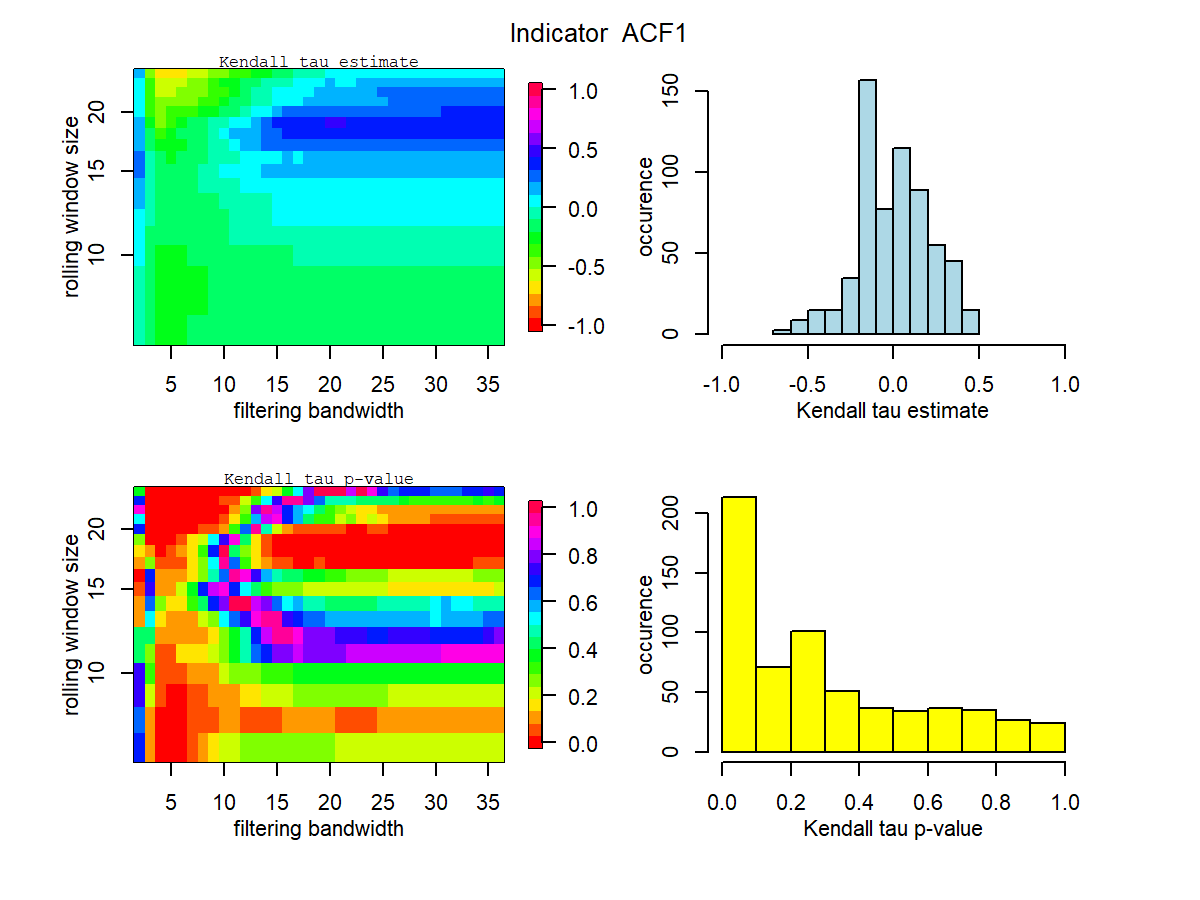


**Figure S20**: Sensitivity analysis for estimating the lag-1 autocorrelations on the short-term sick leave data.


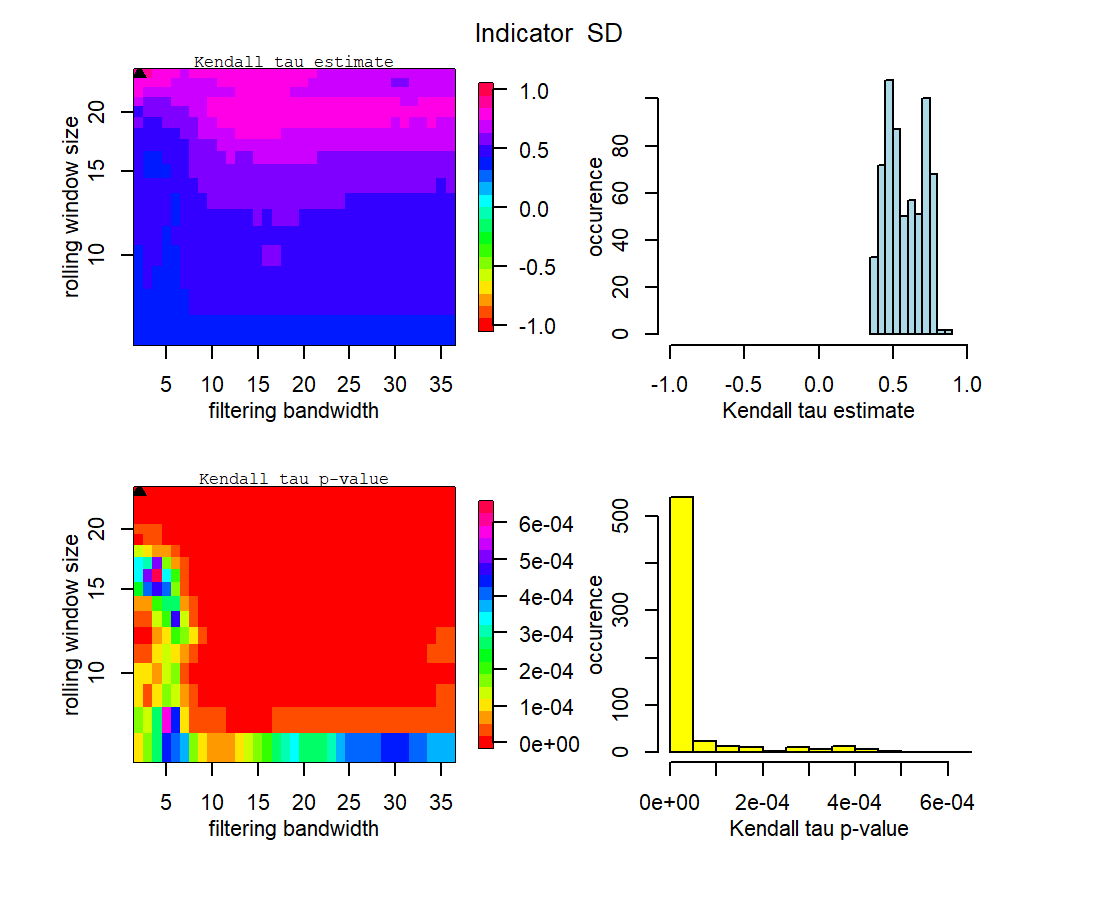


**Figure S21**: Sensitivity analysis for estimating the standard deviations on the short-term sick leave data.


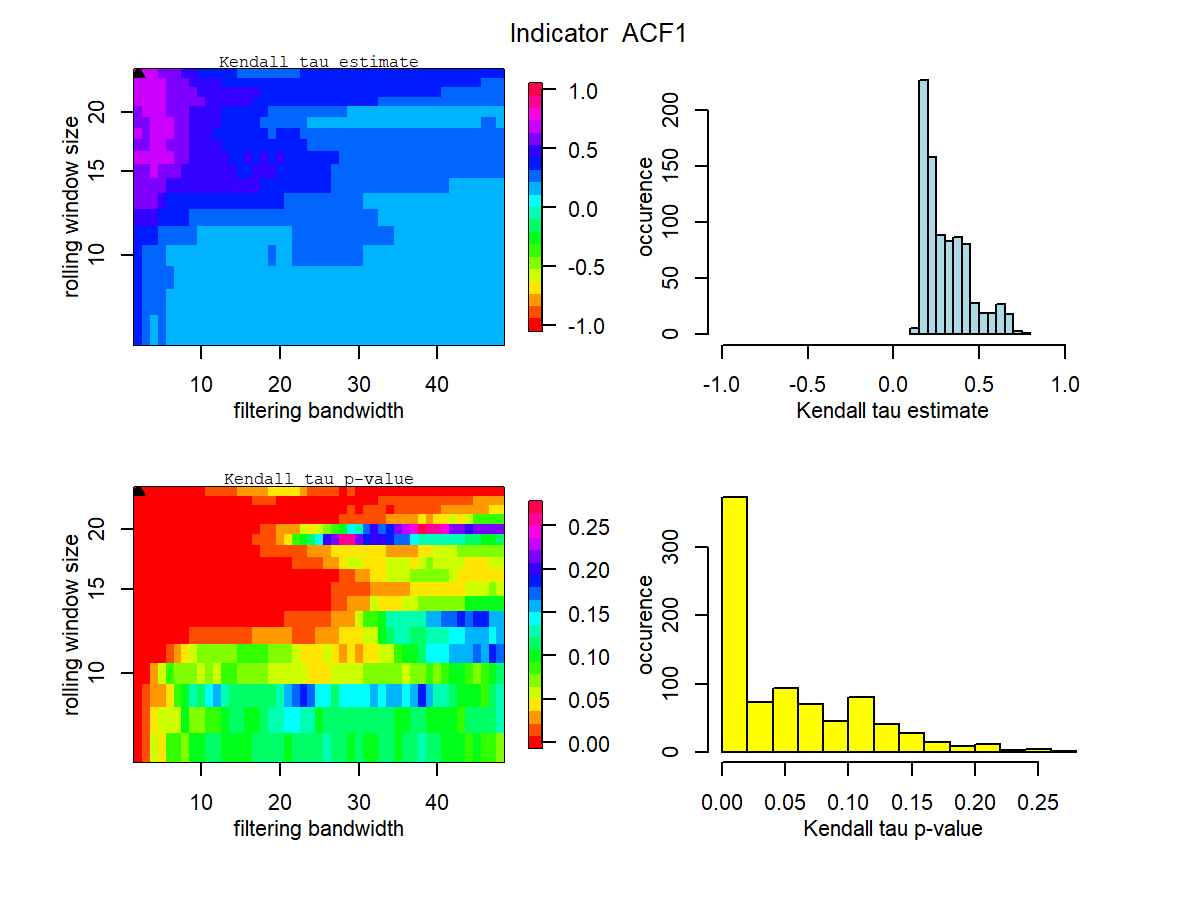


**Figure S22**: Sensitivity analysis for estimating the lag-1 autocorrelations on the long-term sick leave data.


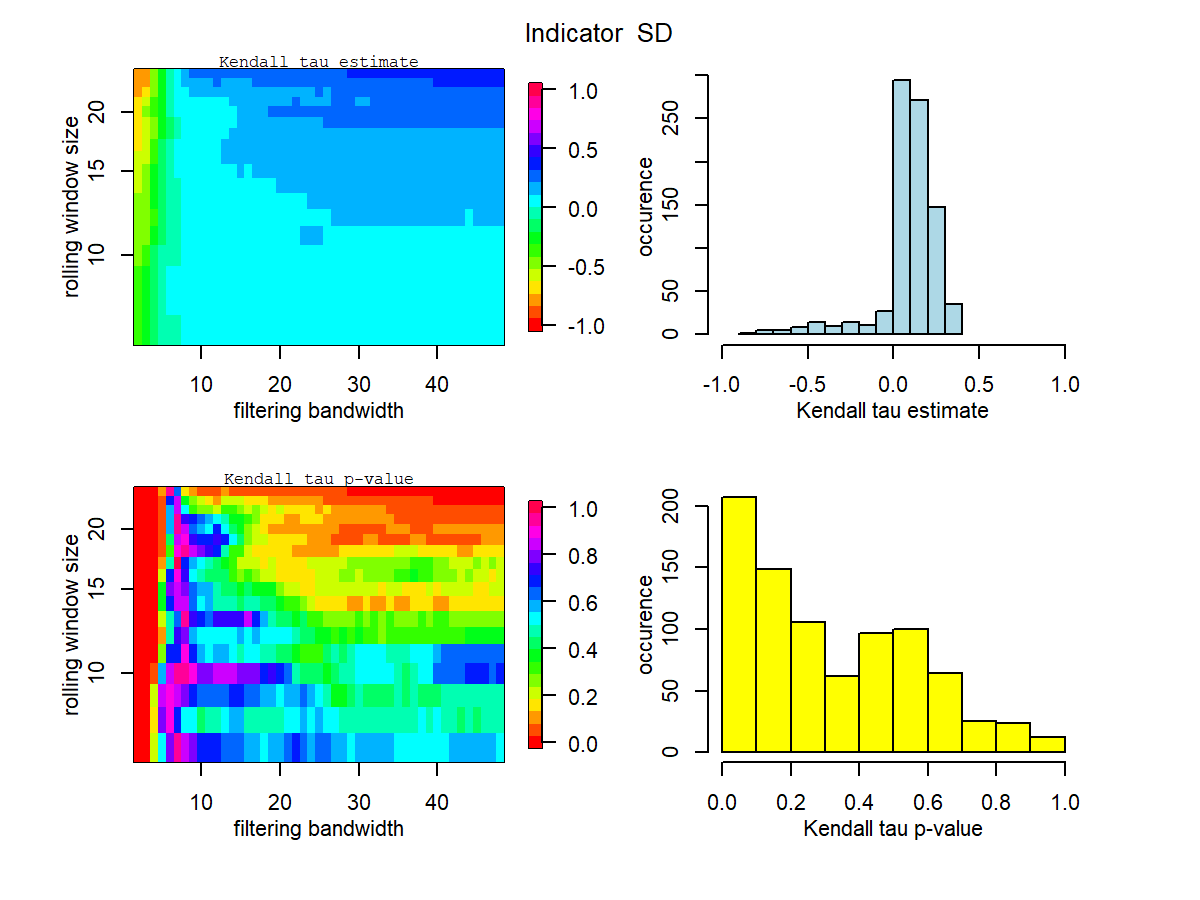


**Figure S23**: Sensitivity analysis for estimating the standard deviations on the long-term sick leave data.

**REFERENCES**

Dessavre, A. G., Southall, E., Tildesley, M. J., & Dyson, L. (2019). The problem of detrending when analysing potential indicators of disease elimination. *Journal of theoretical biology*, *481*, 183-193.
